# Ubiquitin‐specific peptidase 39 promotes human glioma cells migration and invasion by facilitating ADAM9 mRNA maturation

**DOI:** 10.1002/1878-0261.12958

**Published:** 2021-05-02

**Authors:** Yue Xiao, Wenjing Ma, Weiwei Hu, Qianqian Di, Xibao Zhao, Xingyu Ma, Xinyi Chen, Ping Sun, Han Wu, Zherui Wu, Weilin Chen

**Affiliations:** ^1^ Guangdong Provincial Key Laboratory of Regional Immunity and Diseases Department of Immunology School of Medicine Shenzhen University China; ^2^ Department of Neurosurgery Second Affiliated Hospital Zhejiang University School of Medicine Hangzhou China

**Keywords:** ADAM9, glioma, invasion, migration, messenger RNA maturation, USP39

## Abstract

Glioma cells are characterized by high migration and invasion ability; however, the molecular mechanism behind both processes still remains to be investigated. Several studies have demonstrated that ubiquitin‐specific protease 39 (USP39) plays an oncogenic role in various cancer types. Here, we investigated the expression and function of USP39 in patients with glioma. Oncomine database analysis revealed that high USP39 expression was significantly correlated with poor overall survival in patients with glioma. Knockdown of USP39 in U251 and U87 cell lines significantly inhibited their migration and invasion *in vitro*. Gene expression profiling of glioma cells transduced with short hairpin RNA (shRNA) against USP39 revealed that disintegrin and metalloproteinase domain‐containing protein 9 (ADAM9), a molecule previously related to tumor cell migration and invasion, was significantly downregulated. Furthermore, USP39 induced ADAM9 messenger RNA (mRNA) maturation and decreased the expression of integrin β1. Additionally, overexpression of ADAM9 inhibited the migration and invasion of glioma cells caused by USP39 depletion *in vitro*. USP39 promoted the invasion of glioma cells *in vivo* and reduced the overall survival of the mice. Altogether, our data show that USP39 induces mRNA maturation and elevates the expression of ADAM9 in glioma cells and may thus be considered potential target for treating patients with glioma.

## Graphical abstract

Our study shows that ubiquitin‐specific protease 39 (USP39) is highly expressed in human glioma and the high USP39 expression significantly correlated with poor overall survival in patients with glioma. USP39 promotes the migration and invasion of glioma cells *in vitro* and *in vivo*. Mechanically, USP39 induced disintegrin and metalloproteinase domain‐containing protein 9 mRNA maturation and decreased the expression of integrin β1.

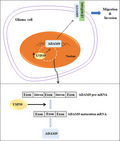

**Abbreviations**
ADAM9: disintegrin and metalloproteinase domain‐containing protein 9
EGF: epidermal growth factor
EV: empty vector
GBM: glioblastoma
GEO: gene expression omnibus
HE: hematoxylin & eosin
IHC: immunohistochemistry
miR: microRNA
MMP: matrix metalloproteinases
mRNA: messenger RNA
PVA: polyvinyl alcohol
RIP: RNA‐binding protein immunoprecipitation
shRNA: short hairpin RNA
shNC: negative control short hairpin RNA
siNC: negative control siRNA
siRNA: small interfering RNA
snRNP: small nuclear RNP
TCGA: The Cancer Genome Atlas
TMA: tissue microarrays
UCH: ubiquitin carboxy‐terminal hydrolases
USP39: ubiquitin‐specific protease 39

## 1 Introduction

Gliomas include well‐differentiated low‐grade astrocytomas, anaplastic astrocytomas, oligodendrogliomas and glioblastoma multiforme (GBM) [1]. GBM is the most commonly occurring primary malignant brain tumor of the central nervous system (CNS) and makes up about 80% of all malignant brain tumors [2]. The most effective therapy for glioma is maximal safe surgical resection followed by radiotherapy with concomitant and adjuvant temozolomide chemotherapy [3]. Despite this multimodality treatment, postoperative recurrence is common and leads to poor outcomes. The median survival rates for glioma are in the range of 14–16 months [4]. In the past decade, although numbers of new molecular features of glioma were discovered, the effective treatment was not significantly improved [3, 5, 6]. Hence, it is essential to identify the major molecular basis for glioma and new biomarkers for ameliorating disease management.

Ubiquitin‐specific peptidase 39 (USP39), also known as 65‐kDa SR‐related protein of the U4/U6·U5 tri‐small nuclear RNP (snRNP), belongs to one member of the deubiquitinating enzymes based on its ubiquitin carboxy‐terminal hydrolases (UCH) domain [7]. However, whether USP39 has the deubiquitinating activity is still a controversial subject. Several studies considered that USP39 lacked the deubiquitinating activity owing to the absence of conserved protease catalytic site residues in the UCH‐domain [7, 8]. Meanwhile, a recent study reported that USP39 could regulate chemo‐radiation resistance in lung cancer cells by deubiquitinating and stabilizing CHK2 protein [9]. Nevertheless, the main biological function of USP39 protein was as a splicing factor to regulate pre‐mRNA maturation. Previous studies have verified that USP39 is involved in specific Aurora B mRNA splicing [8]. USP39 mutations lead to abnormal splicing of the precursor mRNA of retinoblastoma gene, *rb1* [10]. Overexpression of USP39 promoted EGFR mRNA maturation and transcription elongation [11]. Depletion of USP39 inhibited the development of HCC by suppressing FoxM1 pre‐mRNA maturation [12]. Moreover, an oncogenic function of USP39 has been identified in a variety of cancer types, including lung and colon carcinomas [13], human renal cell carcinoma [14], breast cancer [15] as well as other cancers [16, 17, 18]. However, little is currently known about the role of USP39 in GBM.

ADAM9 is a member of disintegrin and metalloproteinase (ADAM) families, which are type I transmembrane proteins. ADAM9 contains several characteristic domains, including cysteine‐rich, propeptide, disintegrin, metalloproteinase, epidermal growth factor (EGF)‐like, transmembrane and cytoplasmic domain [19]. ADAM9 owns shedding and adhesive properties depending on its metalloproteinase and disintegrin domains, respectively [20]. Increased ADAM9 expression has been reported in various cancers, such as hepatocellular carcinoma [21], prostate cancer [22], breast cancer [23, 24], non‐small cell lung cancer [25], skin melanoma [26] and glioma [27]. It was reported that the mRNA levels of ADAM9 were positively associated with histological type and tumor grade in human glioma and could act as a prognostic factor in patients with lower‐grade glioma [27]. However, it is not clear how the expression of ADAM9 is regulated in human glioma.

In this study, we analyzed USP39 expression in human glioma samples from the Oncomine database and its correlation with patient survival. We found that high USP39 expression was positively associated with poor prognosis of glioma patients. We also examined the function of USP39 using U251 and U87 cell lines *in vitro* and mouse xenograft model *in vivo*. Results showed that USP39 promoted the migration and invasion of glioma cells by inducing the pre‐mRNA maturation of ADAM9, a metzincin cell‐surface protease involved in several biological processes such as cell migration and cell–cell interactions in several solid tumors [28]. In addition, USP39 enhanced the invasion ability of glioma cells *in vivo*. This study may provide a new molecular mechanism targeting ADAM9 in the migration and invasion of glioma.

## 2 Materials and methods

### 2.1 Clinical specimens

A total of six human glioma tissues and 67 paraffin‐embedded samples including four normal brain tissues, seven astrocytoma tissues, 21 GBM tissues, 12 oligodendroglioma tissues and 23 metastatic glioma tissues were from the Department of Neurosurgery of the Second Affiliated Hospital, Zhejiang University, School of Medicine (Hangzhou, China). As we previously reported [29] that four normal brain tissues were obtained from patients with cerebral trauma and cerebral hemorrhage, 63 glioma tissues were obtained from the patients who were diagnosed as gliomas by preoperative CT and MRI examinations. Sixty‐seven paraffin‐embedded tissues samples were used for the analysis of the expression levels of USP39 and ADAM9 by immunohistochemistry (IHC). Six frozen samples were subjected to the analysis of the expression levels of USP39 and ADAM9 in human glioma by Western blotting. More information about frozen and paraffin‐embedded samples are shown in Tables S1 and S3. All patients were informed about the experimental details and provided written informed consent. The experimental procedure complied with the Declaration of Helsinki and was approved by the ethics committee of the Zhejiang University (Hangzhou, China) and Shenzhen University (Shenzhen, China).

### 2.2 Cell lines and cell culture

Human GBM U87, U251 cell lines and human embryonic kidney HEK293T cell lines were obtained from the Culture Collection of the Chinese Academy of Sciences (Shanghai, China). All cell lines were cultured in Dulbecco’s modified Eagle medium (DMEM) with 10% FBS.

### 2.3 RNA isolation and quantitative real‐time PCR

Total cells RNAs were isolated by TRIzol reagent (Takara, Beijing, China). Reverse transcription was performed using Reverse Transcriptase M‐MLV (RNase H‐; Takara). Quantitative Real‐Time PCR was performed with Hieff™ qPCR SYBR Green Master Mix (Yeasen Biotech, Shanghai, China) and detected with Analytik Jena qTOWER3 PCR system (Jena, Germany). Data were determined by normalization of expression of GAPDH in each sample. Gene‐specific primer sequences were as following: GAPDH, 5′‐GAGTCAACGGATTTGGTCGT‐3′ and 5′‐GACAAGCTTCCCGTTCTCAG‐3′; USP39, 5′‐GGTTTGAAGTCTCACGCCTAC‐3′ and 5′‐GGCAGTAAAACTTGAGGGTGT‐3′; ADAM9, 5′‐ACTGTGAAAATGGCTGGGCT‐3′ and 5′‐GTATGTAGGTCCACTGTCCACAC‐3′; ADAM9 exon2 unspliced mRNA, 5′‐GCTTTCAACAGACCTC‐3′ and 5′‐GTTTTGAATAGGGCC‐3′; ADAM9 exon2‐exon3 spliced mRNA: 5′‐GCTTTCAACAGACCTC‐3′ and 5′‐GTTCCTTTCCAAGTG‐3′; ADAM9 intron 2, 5′‐TACAGGCATGTGCCA‐3′ and 5′‐TCCACCACAACCGAAA‐3′.

### 2.4 RNA interference

A total of 10^5^ U251 cells were plated in 12‐well cell culture plates, incubated at 37 °C in a 5% CO_2_ incubator for overnight. Twenty nanomolar USP39‐small interfering RNA (siRNA) for U251 cells were added to 100 μL JetPrime buffer, adding 3 μL JetPrime transfection reagents (Polyplus Transfection, New York, NY, USA), vortexing for 10 s and incubating for 10 min at room temperature. The siRNA transfection complexes were added to cell plates. The following sequence was targeted for USP39 siRNA: 5′‐GAUCAUCGAUUCCUCAUUGTT‐3′.

### 2.5 Plasmid, shRNA and transfection

Four shRNA (shRNA1: GATTTGGAAGAGGCGAGATAATCCAAGAGATTATCTCGCCTCTTCCAAATCTTTTTT, shRNA2: GTACTTTCAAGGCCGGGGTTTCAAGAGAACCCCGGCCTTGAAAGTACTTTTTT, shRNA3: GTTGCCTCCATATCTAATCTTTCAAGAGAAGATTAGATATGGAGGCAACTTTTTT, shRNA4: GCCTTCCAGACAACTATGAGATTTCAAGAGAATCTCATAGTTGTCTGGAAGGCTTTTTT) targeting USP39 (NM06590.4) were designed and cloned into lentivirus vector pLent‐4in1 shRNA‐GFP (Vigene Inc., Shandong, China). HEK293T cells were used for packaging lentiviral particles and yielded a high titer (1 × 10^8^/mL). Three days post‐infection, U251 cells with stable knockdown USP39 were screened by GFP and puromycin. For overexpression, the open reading frame of USP39 (NM06590.4) was inserted into pLenti‐EF1a‐FH‐CMV‐RFP‐P2A‐Puro vector (Vigene Inc.). The U87 cells with stable overexpressing USP39 were screened by RFP and puromycin. For transient transfection, the cDNA of USP39 or ADAM9 was cloned into the pCDNA3.1‐HA‐tag vector to generate the USP39 or ADAM9 overexpression plasmid. The overexpression plasmids were verified by sequencing and were transfected into the U87 cells using JetPrime transfection reagents. The efficiency of knockdown was verified by qRT‐PCR and the efficiency of overexpression was determined by western blotting.

### 2.6 Western blotting assay

Lysates from cells and tissues were prepared using RIPA buffer containing a protease inhibitor cocktail (Sigma‐Aldrich, Merck, kGaA, Darmstadt, Germany). Proteins were separated using SDS/PAGE gels and were electrotransferred to nitrocellulose filter membranes (Millipore, Bedford, MA, USA). The membranes were blocked in 5% non‐fat milk and immunoblotted with primary specific antibodies overnight at 4 °C, followed by their respective secondary antibodies. The primary antibodies were used as follows: ADAM9 (#ab186833; Abcam, Cambridge, MA, USA), USP39 (#ab131244; Abcam), integrin β1 (#34971; CST, Danvers, MA, USA), GAPDH (#60004‐1‐Ig; Proteintech, Wuhan, China), β‐Actin (#AA128; Hua An Biotechnology, Hangzhou, China).

### 2.7 Cell migration and invasion assay

A total of 10^5^ U87 cells or U251 cells were plated on a 12‐well plate overnight. The cells were transfected with USP39‐siRNA or scramble‐siRNA for 48 h. The cell monolayers were scratch‐wounded using a sterile micropipette tip to generate a denuded zone of constant width, washed and incubated in DMEM with 2% FBS. The wound sites were photographed. For migration assays, cell suspension (2 × 10^4^/well) was added on top of the Transwell membrane (pore size: 8 μm; Corning Costar, Corning, Bedford, MA, USA) in the upper chamber, and DMEM with 20% FBS (600 μL) was added to the lower chamber. For invasion assays, the upper chamber was coated with BD Biosciences Matrigel (BD Biosciences, Bedford, MA, USA) was added to the upper chamber, and DMEM with 40% FBS (600 μL) was added to the lower chamber. Chambers were incubated at 37 °C and 5% CO_2_ for 24 or 36 h. Finally, cells were fixed with 4% paraformaldehyde (Solarbio, Beijing, China) and stained with Giemsa (Solarbio). Images were acquired under light microscopy from five random fields (×100) in each well. All experiments were conducted in triplicate.

### 2.8 RNA‐binding protein immunoprecipitation assay

RNA‐binding protein immunoprecipitation (RIP) analysis was conducted using the Magna RIP™ kit (Millipore, Billerica, MA, USA) and the USP39 antibody (#23865‐1‐AP; Proteintech) following the manufacturer’s protocol. Co‐precipitated RNA were isolated, purified and subjected to quantitative RT‐PCR analysis.

### 2.9 *In vitro* transcription and splicing assay

*In vitro* transcription and splicing were performed as described previously [30]. Briefly, the ADAM9 mini‐gene was amplified from genomic DNA with primers (5′‐CGCGGATCCGCGAGACCTTTTGCCTGAAGATTTTGTGGT‐3′ and 5′‐CCGCTCGAGCGGCTGAGTCCAAAACAGTCGCTAAGAGCAATGGAT‐3′) by PCR and subcloned into pCDNA3.1 vector using Hieff CloneTM Plus One Step Cloning Kit (Yeasen). The construct was verified by sequencing. The pre‐mRNA transcript of ADAM9 mini‐gene was prepared by *in vitro* transcription reactions using T7 RNA polymerase (P1300; Promega, Madison, WI, USA) according to the manufacturer’s instructions. The splicing extracts were prepared from the whole cell lysates of HEK293T cell with or without USP39. A 25‐μL splicing reaction contained 12.5 mm rATP, 80 mm MgCl_2_, 0.5 mm creatine phosphate, 6.5% polyvinyl alcohol (PVA), 5 ng pre‐mRNA and 10 μL whole cell extracts. Reaction mixtures were incubated at 30 °C for 2 h. The reactions were terminated by adding 10 μL ice‐cold stop mix (5 mm heparin, 5 μL; 87% glycerol, 5 μL), immediately followed by phenol/chloroform extraction and ethanol precipitation. The products were detected by PCR and separated by electrophoresis on a 2.5% agarose gel containing ethidium bromide.

### 2.10 Immunohistochemistry

Deparaffinized tumor tissue sections (5 μm) were subjected to heat‐induced epitope retrieval in citrate buffer (pH 6.0). Then sections were blocked with 5% BSA and incubated with primary antibody included rabbit anti‐USP39 (1 : 100), anti‐ADAM9 (1 : 100) or anti‐integrin β1 (1 : 100) at 4 °C overnight. These slides were incubated with a biotinylated goat anti‐rabbit IgG with the streptavidin‐peroxidase conjugate. Finally, the sections were developed using a 3,3′‐diaminobenzidine (DAB) tetrahydrochloride substrate kit for 3 min at room temperature and then counterstained with hematoxylin. The percentages of positively stained cells were analyzed by using imagej software (National Institute of Health, Bethesda, MD, USA).

### 2.11 Gene expression profiling

The gene expression profiling experiments were performed by SHBIO (Shanghai, China). Briefly, total RNA from U87‐shUSP39 and U87‐shNC cells was isolated using TRIzol reagent. Total RNA was amplified, labeled and purified using Agilent SurePrint G3 Human Gene Expression Microarray 8 × 60 K (ID: 072363) according to the manufacturer’s instructions at Shanghai Biotechnology. Data were extracted with Feature Extraction software 10.7 (Agilent Technologies, Santa Clara, CA, USA). Raw data were normalized by Quantile algorithm, limma packages in r (The R Foundation for Statistical Computing, Vienna, Austria). Limma was validated for differential expression analysis and DAVID was used for functional enrichment analysis.

### 2.12 *In vivo* orthotopic xenografts

Athymic BALB/c nude mice (female, 6‐ to 8‐week‐old) were purchased from Guangdong Medical Laboratory Animal Center (Guangdong, China) and housed in SPF breeding units. Mice were randomly divided into indicated groups. U251‐shNC cells, U251‐shUSP39, U87‐ov‐Con and U87‐ov‐USP39 cells (5 × 10^5^/4 μL) were injected through an entry site at the bregma 2 mm to the right of the sagittal suture and 3 mm below the surface of the skull of anesthetized mice using a stereotactic apparatus (RWD Life Science, Shenzhen, China). Any animal showing symptoms of disease or an impaired general condition after surgery would be excluded from the experiment. Past inoculated of tumor cells 20 or 25 days, mice were systemic perfused with saline solution and 4% paraformaldehyde, and stripped brain tissues were prepared for hematoxylin & eosin (HE) and IHC staining. The animal experimental manipulation was performed according to the National Institute of Health Guide for the Care and Use of Laboratory Animals, with the approval of the Scientific Investigation Board of Science and Technology of Guangdong Province.

### 2.13 Statistical analysis

All experiments assays were conducted at least three times with samples in triplicate. Statistical analysis comparisons between groups were conducted using analysis of Student’s *t*‐tests. A *P*‐value < 0.05 (95% confidence interval) was considered to be statistically significant. Survival curves were estimated for each group, considered separately, using the Kaplan–Meier method and compared statistically using the log‐rank test. A two‐sided chi‐square test and Fisher’s exact test were both used to determine the association between USP39 and ADAM9. Data analysis was conducted using graphpad prism 7 software (GraphPad Software, San Diego, CA, USA).

## 3 Results

### 3.1 USP39 is highly expressed in human glioma

**Fig. 1:**
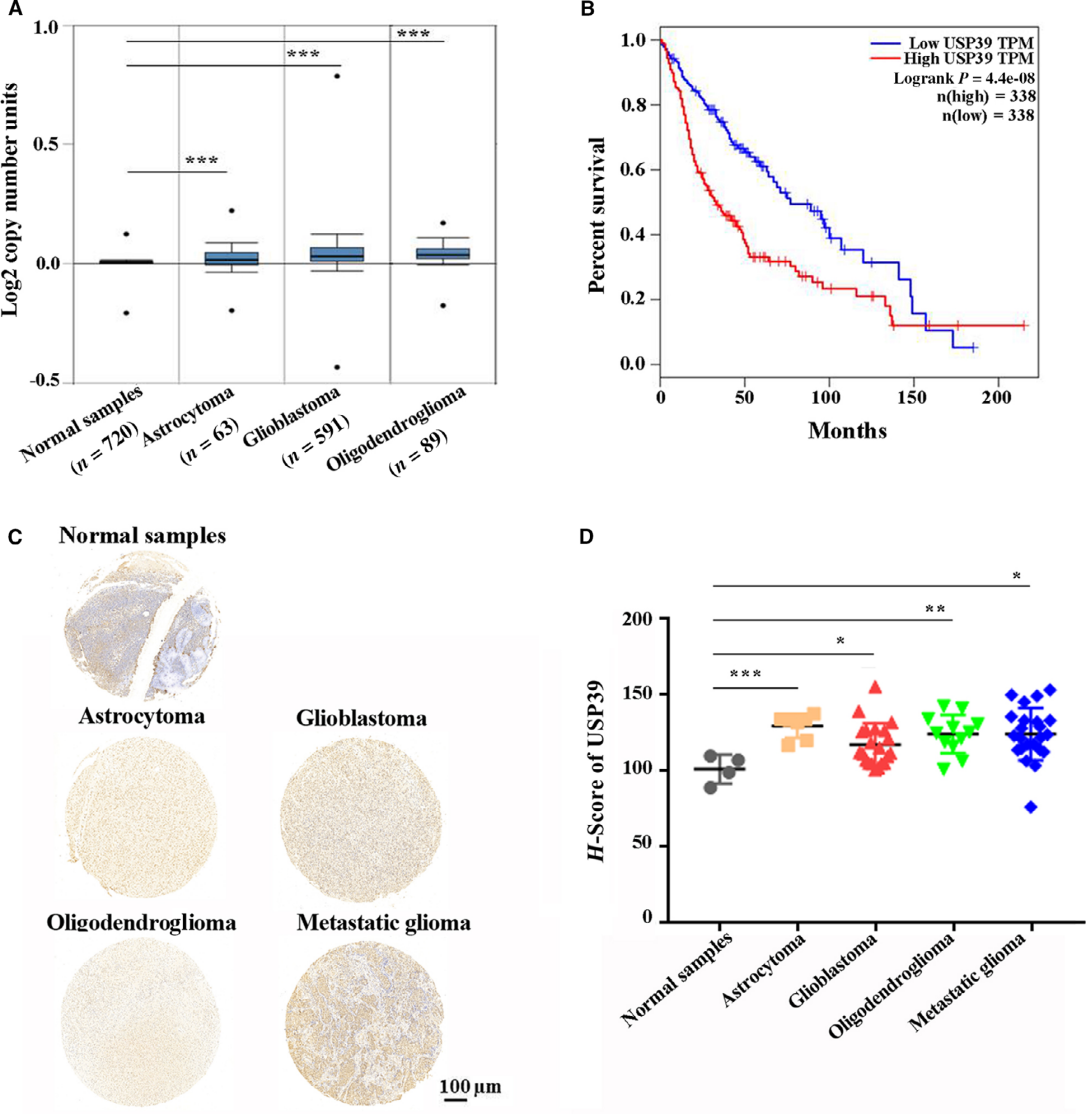
USP39 is highly expressed in human glioma. (A) USP39 copy number in Oncomine database samples grouped by different glioma types. The data are means ± SEM. Significance calculated using Student’s *t*‐test. ***P < 0.001. (B) Kaplan–Meier curve showing the 17‐year survival rate of TCGA samples classified by low‐expressed (*n* = 338) or high‐expressed (*n* = 338) USP39. The *P*‐value was calculated using the log‐rank test. (C) Representative images of IHC staining in human glioma TMA with USP39 antibody. Scale bar: 100 μm. (D) Graphical representation of the H‐score of USP39 in different glioma types from TMA (Normal sample, *n* = 4; Astrocytoma, *n* = 7; GBM, *n* = 21; Oligodendroglioma, *n* = 12; Metastatic glioma, *n* = 23). The data are means ± SEM. Significance calculated using Student’s *t*‐test. *P < 0.05, **P < 0.01, ***P < 0.001.

We first analyzed USP39 expression in different CNS cancer types using the publicly available database Oncomine (https://www.oncomine.org/resource/login.html). Results demonstrated that USP39 was significantly upregulated in all the CNS cancer types, including astrocytoma, GBM, and oligodendroglial tumor (Fig. 1A). No significant differences were observed between the different glioma types. Survival analysis from GEPIA performs [31] (http://gepia.cancer‐pku.cn) revealed that the high expression of USP39 in glioma indicated poor prognosis (Fig. 1B). Whereas, USP39 acts as a component of the tri‐SNP complex, the link between USP39 and overall survival in glioma was not unique. Based on the spliceosome database [32], there were 32 proteins in the tri‐SNP complex of *Homo sapiens* (Fig. S1A). Most of the genes of tri‐snRNP were highly expressed in the GBM samples derived from TCGA database (Fig. S1B). The survival analysis results showed that USP39 ranked at the 11 among the genes which lower expression were associated with a favorable prognosis (Fig. S2). These data indicated that abnormal expression of the proteins of tri‐snRNP complex contributed to the development of glioma. To determine the expression relationship between the USP39 and other components of tri‐snRNP complex, some components of tri‐snRNP complex were randomly selected to be analyzed their correlation with USP39 in the glioma samples from GEPIA databases, and be detected their mRNA levels in the USP39‐overexpressing U87 cells. The results exhibited that most of the genes of tri‐snRNP were positively correlated with USP39 in the glioma samples and upregulated in the USP39‐overexpressing U87 cells (Fig. S3A,B), indicating that USP39 plays a vital role in the tri‐snRNP complex. However, the mechanism in which USP39 regulates the mRNA levels of other tri‐snRNP need further study.

We further validated that the expression levels of USP39 with IHC staining in human glioma tissue microarrays (TMA) from the Department of Neurosurgery of the Second Affiliated Hospital, Zhejiang University School of Medicine (Hangzhou, China; Fig. 1C). The H‐score of USP39 expression levels was higher in astrocytoma, GBM, oligodendroglioma and metastatic glioma than in normal brain tissues (Fig. 1D). Taken together, these results suggested that high USP39 expression was positively correlated with the occurrence of glioma.

### 3.2 Knockdown of USP39 by siRNA inhibits migration and invasion of human glioma cells

**Fig. 2:**
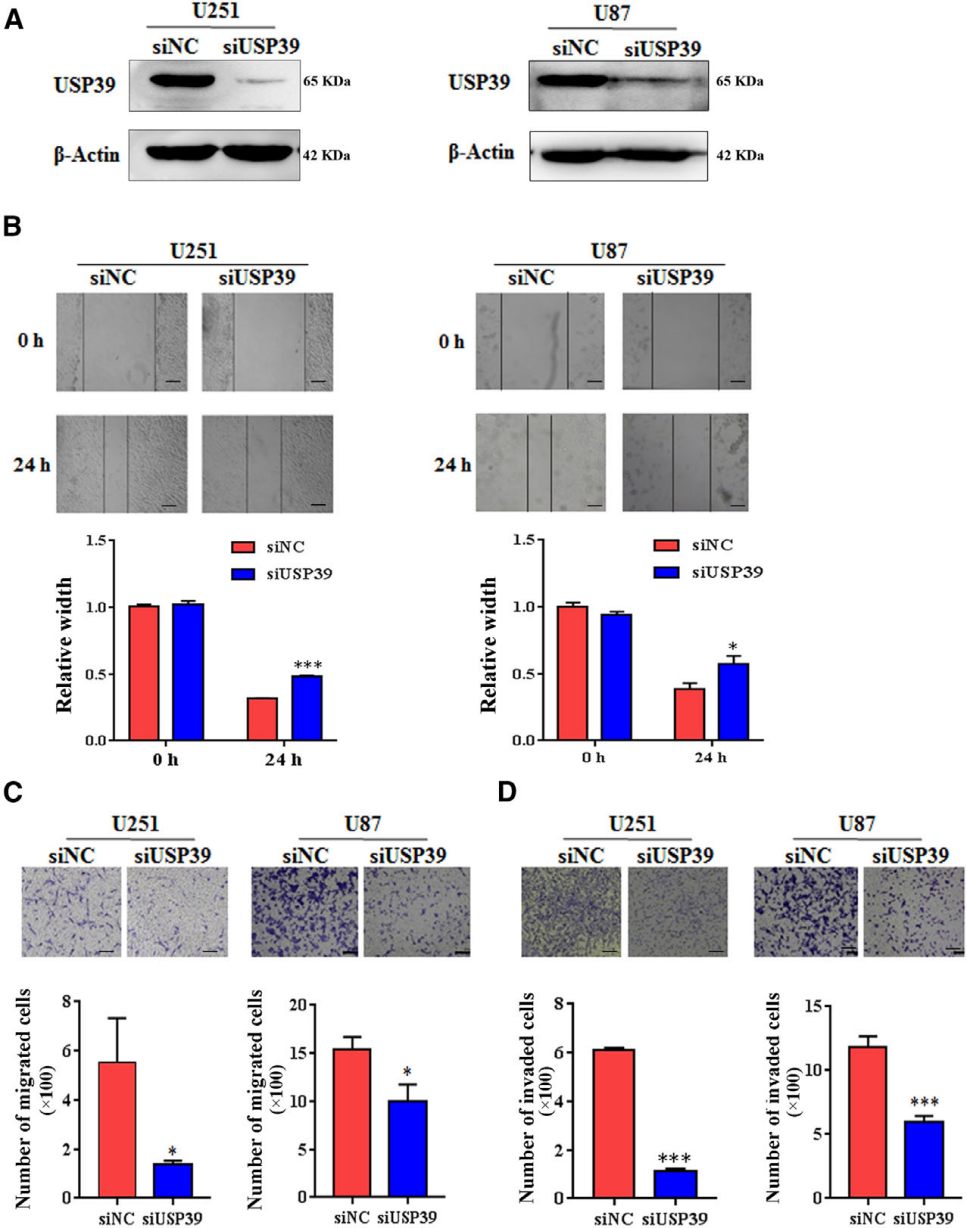
Knockdown expression of USP39 suppresses the migration and invasion of glioma cells *in vitro*. (A) Analysis of USP39 expression in U251 and U87 cells treated with negative control siRNA (siNC) or USP39‐siRNA (siUSP39) examined by western blotting. (B) A wound healing assay was used to evaluate the migration ability of U251 and U87 cells transfected with siNC or siUSP39. Representative digital pictures were taken at 0 h and 24 h. Scale bar: 50 μm. Bar graphs exhibited the relative width of gaps. (C) Transwell migration assay of U251 and U87 cells transfected with siNC or siUSP39. The average number of migratory cells was counted in triplicate. Scale bar: 50 μm. (D) Transwell invasion assay of U251 and U87 cells. The average number of invaded cells was counted in triplicate. Scale bar: 50 μm. All data are presented as means ± SD. Significance calculated using Student’s *t*‐test. *P < 0.05, ***P < 0.001. Representative data are from three independent experiments.

To detect the role of USP39 in the development of glioma, we knocked down the expression of USP39 in glioma cells with siRNA. The human glioma cell lines U251 and U87 were selected as the targeted cells for knocking down the USP39 expression. The siRNA targeting the coding region of USP39 was designed and tested in U251 and U87 cells. The knockdown efficiency of USP39 was detected by Western blot (Fig. 2A). Since metastasis and recurrence represent the main malignant characteristics of malignant glioma. We examined the migration and invasion ability of U251 and U87 transfected with USP39 siRNA. Under the cell culture media with 2% FBS or without FBS, USP39 had no effect on glioma cells proliferation (data not shown). Thus, the migration and invasion tests were performed under the cell culture media with 2% FBS or without FBS to exclude the interference induced by cell proliferation. In a scratch‐wound assay, scratch widths were measured and imaged at 24 h after initial wounding. As shown in Fig. 2B, the width of wound area of U251‐siUSP39 and U87‐siUSP39 significantly increased in 24 h. In cell migration (Fig. 2C) and invasion (Fig. 2D) assays, the number of migrated and invaded cells was reduced by almost 60% in U251 cells transfected with USP39 siRNA. Additionally, in U87 cells, knockdown of USP39 also significantly inhibited the cells migration and invasion. These findings indicated that knockdown expression of USP39 could inhibit migration and invasion of glioma cells *in vitro*.

### 3.3 Overexpression of USP39 promotes migration and invasion of human glioma cells

**Fig. 3:**
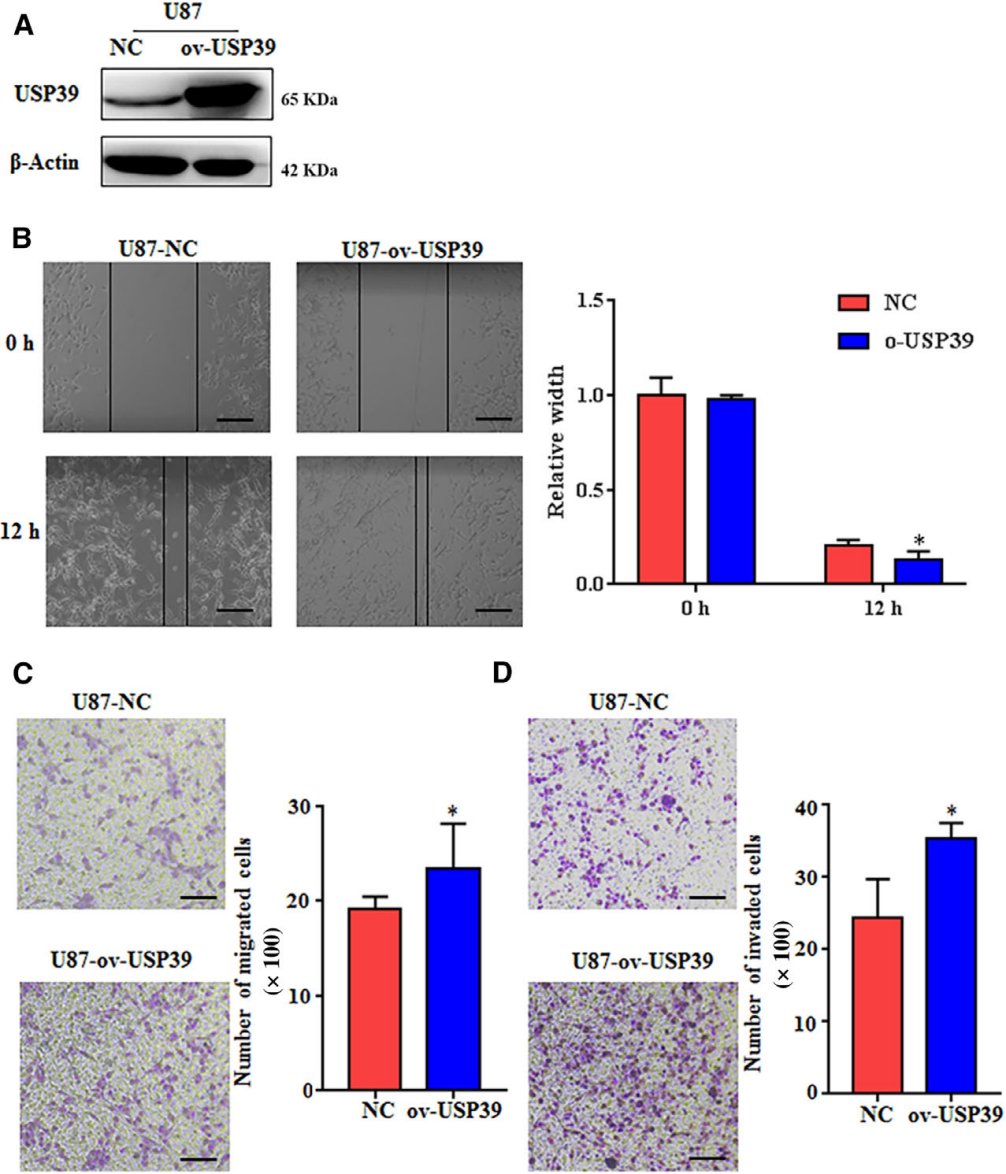
Overexpression of USP39 promotes the migration and invasion of glioma cells *in vitro*. (A) Analysis of USP39 expression in U87 cells transfected with control plasmid (NC) or HA‐tag USP39 (ov‐USP39) plasmid determined by western blotting. (B) A wound healing assay was used to evaluate the migration ability of U251 and U87 cells transfected with NC or ov‐USP39. Scale bar: 100 μm. Bar graphs exhibit the relative width of gaps. (C) Transwell migration assay of U87 cells transfected with NC or ov‐USP39. The average number of migratory cells was counted in triplicate. Scale bar: 100 μm. (D) Transwell invasion assay of U87 cells transfected with NC or ov‐USP39. The average number of invaded cells was counted in triplicate. Scale bar: 50 μm. All data are presented as means ± SD. Significance calculated using Student’s *t*‐test. *P < 0.05, **P < 0.01. Representative data are from three independent experiments.

To further validate the function of USP39 in the development of human glioma, we constructed a USP39 expression plasmid and transfected it into glioma cells. As that the expression levels of USP39 in U87 cells was lower than that in U251 cells (Fig. S4), the U87 cells were selected as the targeted cells for USP39 overexpression. Western blotting analysis showed that the USP39 was highly expressed in U87 cells containing the USP39‐coding plasmid (Fig. 3A). In a scratch‐wound assay, overexpressed USP39 promote the “healing” of the wound gap at 12 h after initial wounding (Fig. 3B). *In vitro* cell migration assays, the migrated cells were increased about 25% in the U87‐ov‐USP39 cells compared to U87‐NC cells (Fig. 3C). Meanwhile, overexpression of USP39 promotes ~ 0.5‐fold increase in U87 cells invasion when compared to negative control (NC) cells (Fig. 3D).

### 3.4 USP39 expression positively correlates with ADAM9 expression in human glioma

**Fig. 4:**
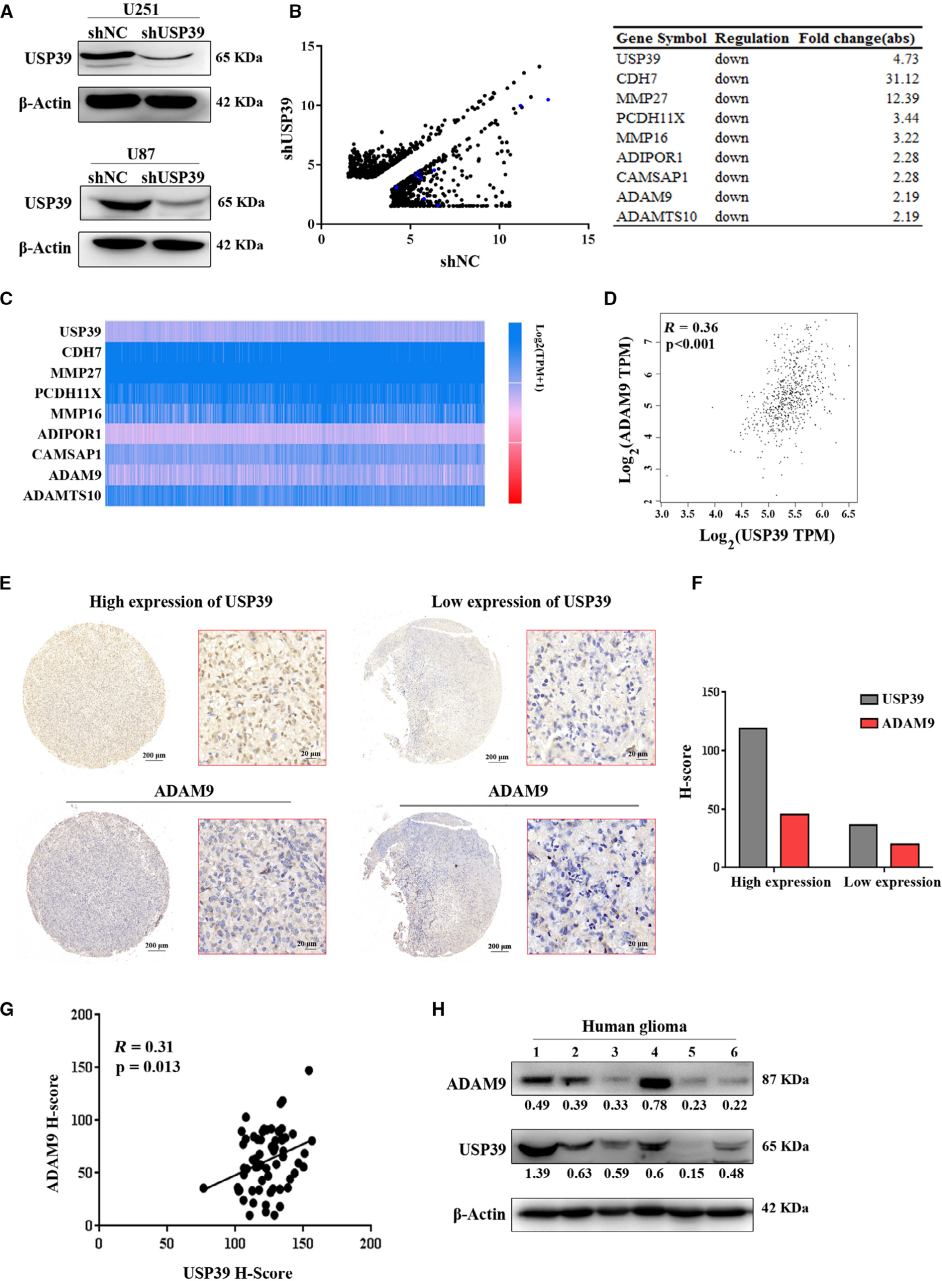
USP39 expression positively correlates with ADAM9 expression in human glioma. (A) The U251 and U87 cells were transfected with lentiviruses encoding USP39‐targeting or negative control shRNA (shUSP39 or shNC), the protein expression levels of USP39 in the cells were analyzed by western blotting. (B) The total RNA from U87‐shUSP39 and U87‐shNC cells was isolated to perform gene expression profiling. Scatterplots display differential gene expression between U87‐shUSP39 and U87‐shNC (fold change > 2, *n* = 1242) from the data of gene expression microarray. The blue point chart and table on the right exhibit the downregulated genes related to the migration and invasion of cancer cells in the U87‐shUSP39 cells. Significance was calculated using Student’s *t*‐test. *P* < 0.05. (C) Expression analysis of the above downregulated genes in human glioma from TCGA samples. (D) Statistical analysis of the correlation analysis between USP39 and ADAM9 expression of TCGA glioma samples from GEPIA databases. The data were examined by calculating Pearson’s correlation coefficients (*n* = 681, *P* < 0.001, *R* = 0.36). (E) IHC images of USP39 (upper) and ADAM9 (lower) in tissue microarray (TMA) slides of paraffin‐fixed human glioma tissues. Scale bar: 200 or 20 μm (as indicated in the picture). (F) Quantification of USP39 and ADAM9 protein levels for the cases shown in (E). (G) Correlation between USP39 and ADAM9 protein expression in human glioma TMA. The data were examined by calculating Pearson’s correlation coefficients (*n* = 63, *P* = 0.013, *R* = 0.31). (H) Protein level of USP39 and ADAM9 in human glioma tissues detected by western blotting, and ratios of gray scale showing the difference in the picture.

To find the target genes of USP39, we infected U251 cells and U87 cells with lentiviruses encoding USP39‐targeting or negative control‐shRNA (shUSP39 or shNC) and confirmed effective protein knockdown by western blotting (Fig. 4A). Gene expression microarray was performed to analyze the expression differences between U87‐shUSP39 and U87‐shNC cells. The data of gene expression microarray have been deposited in NCBI’s Gene Expression Omnibus (GEO) and are accessible through GEO Series accession number GSE159823. Gene Ontology analysis of the differentially expressed genes in U87 cells revealed that genes associated with cellular processes and biology processes were significantly affected by USP39 knockdown (Fig. S5), and the data corresponded with the downregulated abilities of migration and invasion of U87 cells with USP39 siRNA. Analysis of gene expression microarray data indicated that, when the level of USP39 mRNA was decreased more than four‐fold by infection with shRNA, there were 1242 genes whose expression decreased or increased more than two‐fold (Fig. 4B). Furthermore, we analyzed the differentially expressed genes regulating migration and invasion of cancer cells, including the genes of cell adhesion molecules, integrins, epithelial‐mesenchymal transition biomarkers, matrix metalloproteinases, ADAM (a disintegrin and metalloproteinase) family and others. The results showed that twenty‐one genes differentially expressed in the U87‐shNC cells and U87‐shUSP39 cells (Table S2). Then, we analyzed the expression levels of the eight downregulated genes following USP39 knockdown, and the correlation between their expression and USP39 expression in TCGA glioma samples [33]. The results indicated that the positive correlation between ADAM9 and USP39 expression was the most significant (Fig. 4B–D, Fig. S6). TMA of 63 human gliomas were analyzed to further confirm the expression correlation of USP39/ADAM9 axis. Representative images of IHC assay from TMA were shown in Fig. 4E. The H‐score was validated for the assessment of protein expression level. As shown in Fig. 4F,G, the expression of USP39 was positively correlation with ADAM9 expression in TMA. Furthermore, six fresh human glioma tissues were used to detect the expression levels of USP39 and ADAM9 by Western blotting. The result showed that, in the samples with high USP39 expression, the expression levels of ADAM9 were relatively high (Fig. 4H). The detailed information of six fresh samples was showed in Table S3. Collectively, USP39 expression positively correlates with ADAM9 expression in human glioma.

### 3.5 Ectopic expression of ADAM9 rescues the impaired abilities of migration and invasion of U87 cells induced by USP39 knockdown

**Fig. 5:**
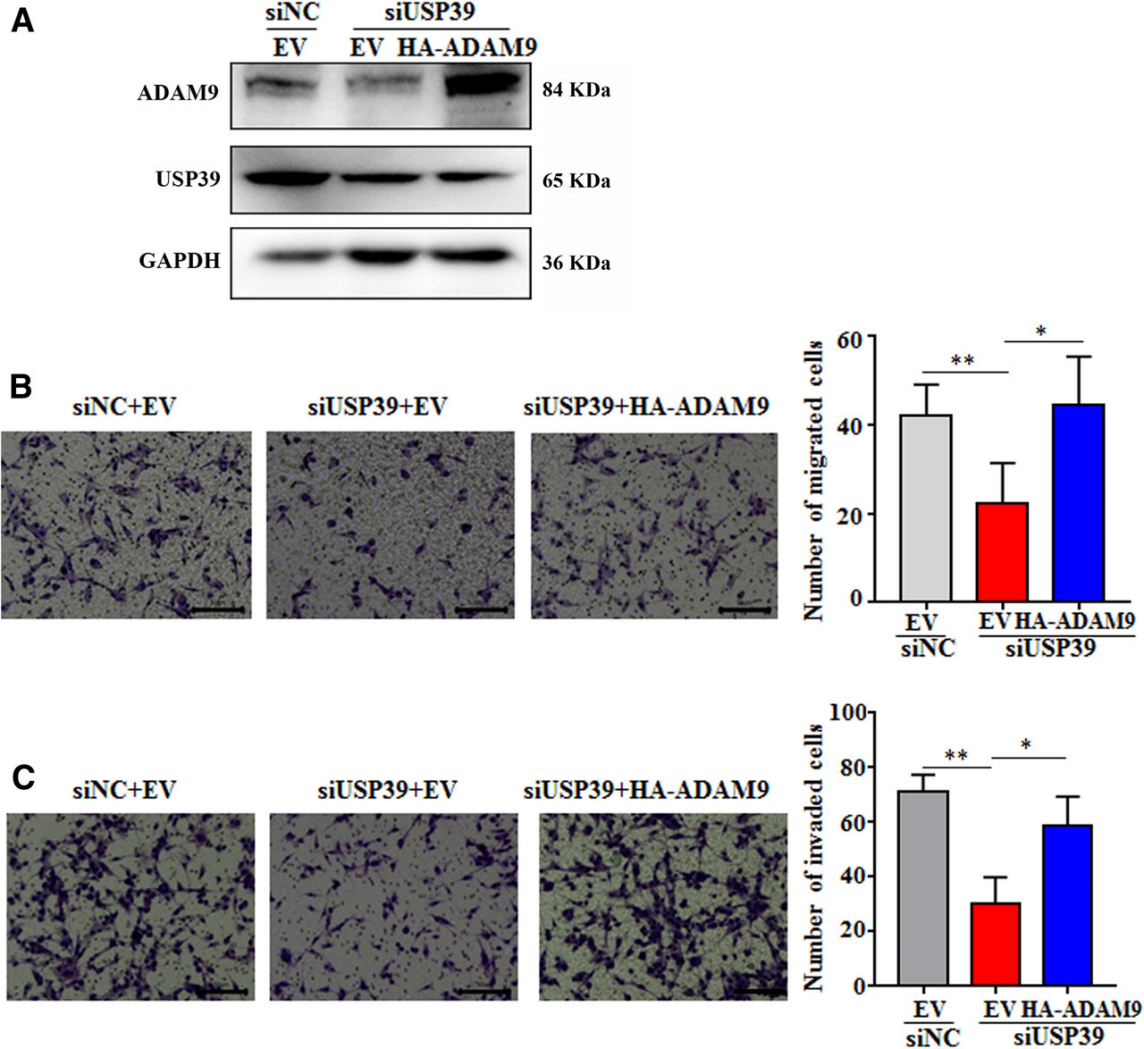
Overexpressed ADAM9 rescues the impaired abilities of migration and invasion of U87 cells induced by USP39 depletion. (A) Analysis of ADAM9 expression in U87 cells transfected with siNC or siUSP39 plus ADAM9 overexpressed (HA‐ADAM9) plasmid or EV plasmid determined by western blotting. (B) Transwell migration assay of U87 cells. Migrating cells were observed under a light microscope. The average number of migratory cells was counted in triplicate. Scale bar: 100 μm. (C) Transwell invasion assay of U87 cells. The average number of migratory cells was counted in triplicate. Scale bar: 100 μm. All data are presented as means ± SD. Significance calculated using Student’s *t*‐test. *P < 0.05, **P < 0.01. Representative data are from three independent experiments.

To further investigate whether USP39 promotes migration and invasion of glioma cells by regulating the expression of ADAM9, the U87 cells with USP39 siRNA or negative control‐siRNA were co‐transfected with an ADAM9‐overexpression plasmid or negative control empty vector (EV). There was about 26% cells transfected with GFP‐tagged ADAM9 (Fig. S7). The overexpression level of ADAM9 was identified by Western blotting (Fig. 5A). In cell migration assays, over‐expressed ADAM9 in the U87 cells expressing USP39 siRNA rescued cell migration to the level seen in the U87 cells expressing NC siRNA (Fig. 5B). Besides, in cell invasion assays, ADAM9 overexpression also rescued the decreased invasion in U87 cells by USP39 knockdown (Fig. 5C). Meanwhile, to further confirm the rescue effect of ADAM9 on the USP39‐regulated migration and invasion of glioma cells, we knock down the expression of ADAM9 in USP39‐overexpressed U87 cells. The results showed that the numbers of cell migration and invasion in the U87‐ov‐USP39 cells transfected with ADAM9 siRNA were less than that in the U87‐ov‐USP39 cells transfected with negative control‐siRNA (Fig. S8). Thus, the above results demonstrated that USP39 promoted migration and invasion of glioma cells in a manner dependent upon the expression of ADAM9.

### 3.6 USP39 regulates ADAM9 protein expression through inducing ADAM9 mRNA maturation

**Fig. 6:**
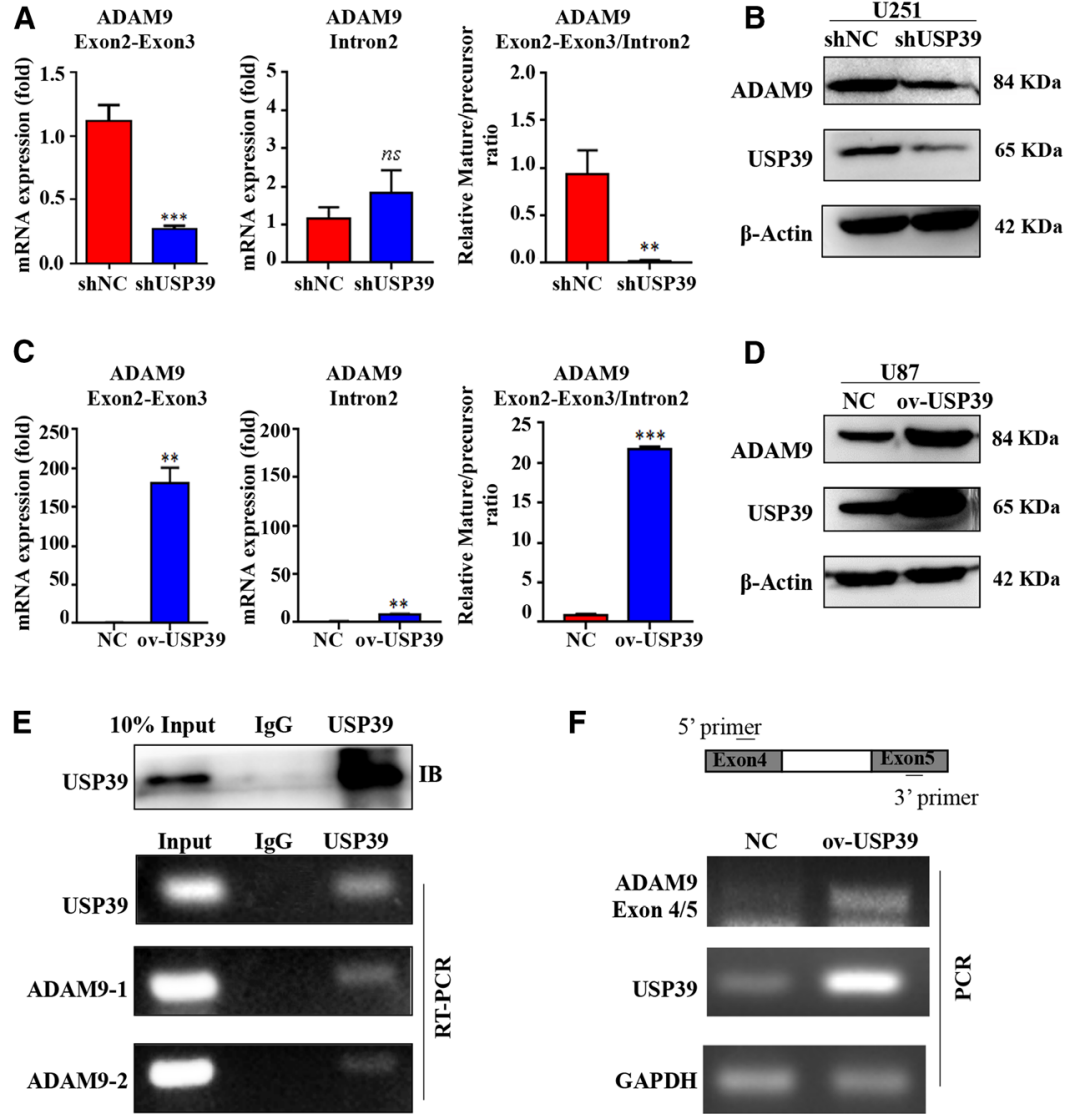
USP39 promotes the ADAM9 pre‐mRNA splicing. (A) qRT‐PCR analysis exhibiting relative mRNA levels of mature and precursor ADAM9 RNA transcripts and the ratio in U251 cells treated with USP39 siRNA relative to controls. (B) Western blotting analysis of USP39 and ADAM9 expression levels in U251 cells infected with Control‐shRNA‐expressing lentiviruses (shNC) or USP39‐shRNA‐expressing lentiviruses (shUSP39). (C) qRT‐PCR analysis showing relative mRNA levels of mature and precursor ADAM9 RNA transcripts and the ratio in U87 cells transfected with USP39 overexpressed plasmid relative to controls. (D) Western blotting analyzed USP39 and ADAM9 expression in U87 cells transfected with control plasmid (NC) or USP39 overexpressed plasmid (ov‐USP39). (E) RIP assay was conducted using anti‐USP39 (with IgG as control), and RT‐PCR were used to determine the mRNA level of ADAM9 in the immunoprecipitated complex. (F) Schematic model (top) representation of a region of ADAM9 gene used to prepare DNA template to synthesize pre‐mRNA substrate for *in vitro* splicing assay. Bottom: PCR fragment amplified with 5′ and 3′ primers using the DNA template from spliced product using nuclear extract from HEK293T cells carrying control vector or USP39 overexpressed plasmid. All data are presented as means ± SD. Significance calculated using Student’s *t*‐test. **P < 0.01, ***P < 0.001. Representative data are from three independent experiments.

USP39 has been reported to be involved in pre‐mRNA splicing of several genes. We then examined whether USP39 had a function on posttranscriptional regulation of ADAM9 mRNA splicing. However, according to the Ensembl database, ADAM9 have five alternative transcripts, only one transcript could yield a protein isoform and be observed in this work. The region from exons 2 to 3 in the transcript (ENST00000487273.7) was selected to design specific primers excluding the intron 2 to distinguish ADAM9 mature mRNA, and the intron 2 in the transcript was selected to design specific primers to distinguish ADAM9 precursor mRNA. The ratio of mature to precursor ADAM9 mRNA was calculated for estimating the ADAM9 mRNA maturation. As shown in Fig. 6A, the relative mature mRNA levels of ADAM9 was significantly downregulated in the U251 cells expressing USP39 shRNA, while, the intron 2 mRNA levels of ADAM9 did not change upon USP39 depletion. Reasonably, the ratio of mature to precursor ADAM9 mRNA levels and the protein expression levels of ADAM9 were all dramatically reduced (Fig. 6A,B). Accordingly, overexpression of USP39 in U87 cells promoted the ADAM9 mRNA maturation and protein expression (Fig. 6C,D). To further confirm the molecular mechanism of USP39 regulating ADAM9 mRNA maturation, an RIP assay was performed on extracts prepared from U87 using USP39 antibody. The results of RT‐PCR demonstrated that ADAM9 expression was significantly enriched in USP39 pull downs compared with IgG controls (Fig. 6E). Besides, an *in vitro* splicing assay was performed to examine the effect of USP39 on splicing. First, we amplified a mini‐gene of ADAM9 containing two exons and one intron (Fig. 6F) by PCR using the genomic DNA of U87 as template, and cloned the PCR fragment into an empty vector (EV). Then, the linear recombinant plasmid containing the mini‐gene of ADAM9 was used to synthesize the ADAM9 precursor mRNA in an *in vitro* transcription. The obtained ADAM9 precursor mRNA transcript was incubated in a splicing reaction with HEK293T cell nuclear extract for 2 h. PCR demonstrated that more mature mRNA products were acquired by adding USP39 (Fig. 6F). Furthermore, to determine whether USP39 could interact with ADAM9 and regulate the ubiquitination of ADAM9, we co‐transfected Myc‐tagged USP39, Flag‐tagged ADAM9 with or without HA tagged Ub plasmids into HEK293T cells; co‐immunoprecipitation experiments revealed that USP39 failed to interact with ADAM9 at the protein level (Fig. S9A) and overexpressing USP39 had no effect on the polyubiquitination of ADAM9 (Fig. S9B). These data suggested that USP39 regulated ADAM9 expression at least in part by promoting its pre‐mRNA splicing.

### 3.7 Knockdown of USP39 expression inhibits the progression of glioma *in vivo*

**Fig. 7:**
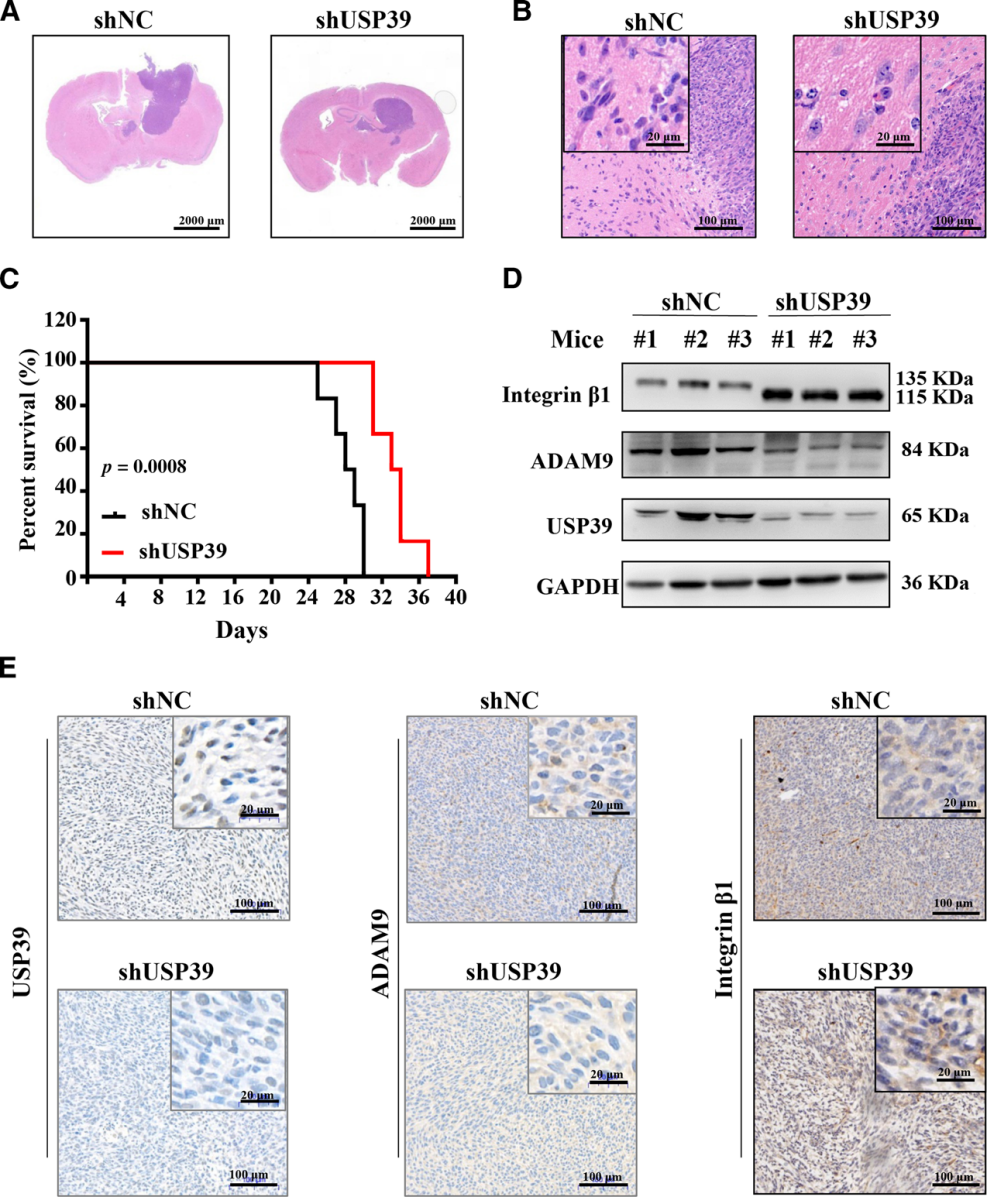
Knockdown expression of USP39 inhibits the growth and invasion of U251 cells *in vivo*. The U251 cells expressing USP39 shRNA or negative control shRNA were intracranially implanted into BALB/c nude mice. (A, B) Hematoxylin‐eosin (HE) staining of orthotopic xenografts derived from the indicated U251‐shUSP39 cells and control in nude mice (*n* = 3/group). Representative images are shown. Scale bar: 2000, 20 or 100 μm (as indicated in the picture). (C) Kaplan–Meier analysis of survival for tumor‐bearing mice implanted with U251‐shUSP39 cells and control (*n* = 6/group). Log‐rank test: *P* < 0.001. (D) Western blotting analysis of USP39, ADAM9 and integrin β1 protein levels in tumor tissues. (E) IHC staining of USP39, ADAM9 and integrin β1 levels in xenograft sections from U251‐shNC and U251‐shUSP39 groups. Representative images are shown. Scale bar: 20 or 100 μm (as indicated in the picture). Representative data are from three independent experiments.

To determine the effects of USP39 on the progression of glioma *in vivo*, the U251 cells expressing USP39 shRNA or negative control shRNA were intracranially implanted into BALB/c nude mice. The soft tumor‐bearing brain tissues were completely stripped from the skull region of head and were used for histological analysis. As shown in Fig. 7A, the tumor area in U251‐shUSP39‐bearing brain was less than that in U251‐negative control short hairpin RNA (shNC)‐bearing brain at 20 days after cell injection. Histological examination revealed that U251 cells treated with shUSP39 orthotopic xenografts were less invasive than the same cell treated with shNC (Fig. 7B). Furthermore, we observed the survival of the mice carrying established intracranial U251 glioma. As shown in Fig. 7C, loss of USP39 in U251 cells significantly prolonged survival of the tumor‐bearing mice (*P* < 0.001). At 30 days after U251‐inoculated cells, all of the mice in the U251‐shUSP39 group were alive, whereas all of the mice in the U251‐shNC group were dead. By 37 days post inoculation of U251 cells, the last alive mouse in the U251‐shUSP39 group was dead. Finally, the expression levels of USP39/ADAM9 in the brain tumor were detected by western blotting and IHC analysis. In the U251‐shUSP39‐bearing tumor, the expression levels of USP39 and ADAM9 were downregulated compared with that in the U251‐shNC‐bearing tumor (Fig. 7D,E). To understand in greater depth the role of the USP39/ADAM9 axis in glioma cell migration and invasion, we detected the expression level of integrin β1, an ADAM9‐interacting protein, in U251 and U87 cells infected with USP39‐shRNA‐expressing lentiviruses. Western blotting and flow cytometry assay revealed that the expression level of integrin β1 were upregulated in U251‐shUSP39 cells relative to controls (Fig. S10A,B). The results demonstrated that knockdown of USP39 could induce the upregulated integrin β1 expression on U251 cell surface. Therefore, we further examined the relationship between USP39/ADAM9 axis and integrin β1 *in vivo*. As shown in Fig. 7D,E, the integrin β1 expression was increased in the U251‐shUSP39‐bearing tumor compared with that in the U251‐shNC‐bearing tumor. These results suggested that loss of USP39 expression inhibits the invasion of glioma cells *in vivo*.

### 3.8 Overexpressed USP39 promotes the progression of glioma *in vivo*

**Fig. 8:**
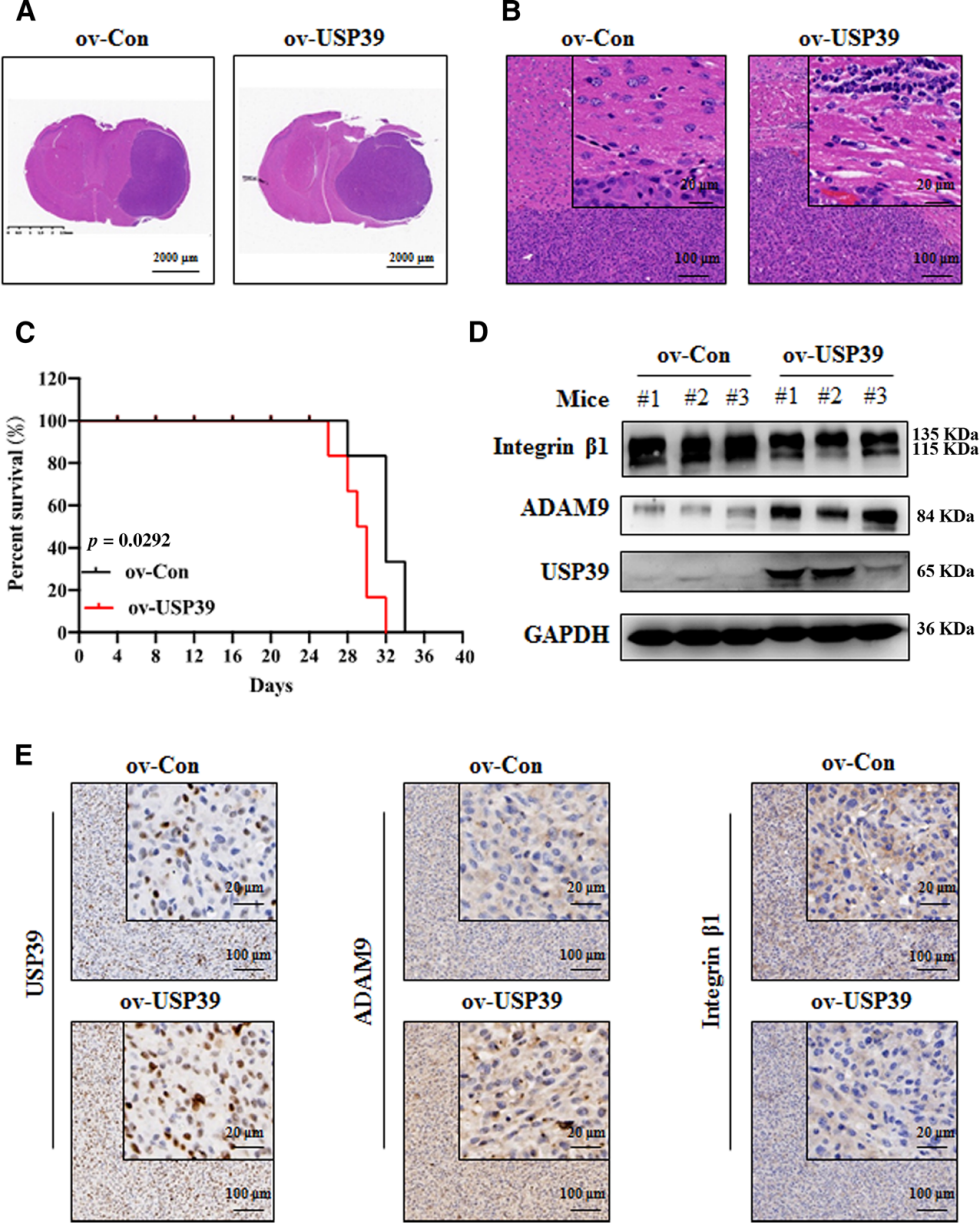
Overexpression of USP39 promotes the growth and invasion of U87 cells *in vivo*. (A, B) Representative images of HE staining of orthotopic xenografts obtained from the indicated U87‐ov‐USP39 cells and control in nude mice (*n* = 3/group). Scale bar: 2000, 20 or 100 μm (as indicated in the picture). (C) Kaplan–Meier analysis of survival for tumor‐bearing mice implanted with U87‐ov‐USP39 cells and controls (*n* = 6/group). Log‐rank test: *P* < 0.05. (D) Western blotting analysis of USP39, ADAM9 and integrin β1 protein levels in tumor tissues. (E) IHC staining of USP39, ADAM9 and integrin β1 levels in xenograft sections from U87‐ov‐Con and U87‐ov‐USP39 groups. Representative images are shown. Scale bar: 20 or 100 μm (as indicated in the picture). Representative data are from three independent experiments.

The U87 cells infected with USP39‐expressing lentiviruses were intracranially implanted into the brains of BALB/c nude mice. Histological examination revealed that the tumor area in U87‐ov‐USP39‐bearing brain was larger than that in U87‐ov‐Con‐bearing brain at 25 days after cell injection (Fig. 8A); U87‐ov‐USP39 orthotopic xenografts tended to be more invasive than U87‐ov‐Con (Fig. 8B). The survival curve showed that overexpression of USP39 in U87 cells reduced the survival of the tumor‐bearing mice (*P* < 0.05; Fig. 8C). In addition, we detected the expression levels of USP39, ADAM9 and integrin β1 in the brain tumor. As shown in Fig. 8D,E, in the U87‐ov‐USP39‐bearing tumor, the expression levels of USP39 and ADAM9 were upregulated but the expression levels of integrin β1 were downregulated compared with those in the U87‐ov‐Con‐bearing tumor. Collectively, these results demonstrated that overexpressed USP39 promotes glioma cells invasion *in vivo*.

## 4 Discussion

Despite advances in the diagnosis and treatment of glioma, the prognosis of the disease is still poor, especially for patients with malignant and invasive gliomas. The highly invasive characteristic contributes to the difficulty to complete resection of the tumor, causing significant neurologic morbidity and mortality [34, 35, 36]. Understanding the molecular mechanism of glioma migration and invasion could help to advance the diagnosis and therapy of glioma. In the present study, we identified an oncogenic role for USP39 in human glioma and confirmed its function in the migration and invasion of glioma. The phenotypes in this study are similar to the previous study [18], which showed that USP39 promotes glioma progression by inducing TAZ mRNA maturation. That study first revealed the functional role of USP39 in the development of glioma *in vitro* and *in vivo*. Mechanically, those authors demonstrated that loss of USP39 led directly to a decreased TAZ mRNA maturation detected by qRT‐PCR and RIP [18]. In our study, not only qRT‐PCR and RIP techniques but also *in vitro* splicing transcription and splicing assay were established to demonstrate a novel molecular mechanism of USP39 in regulating the development of glioma. The mechanism is that USP39 directly bind with ADAM9 mRNA and promote its pre‐mRNA maturation, following the altered expression and activity of integrin β1. Collectively, our findings are similar to the oncogenic role for USP39 in various tumors. These data provide new insights into the involvement of USP39 and ADAM9 in glioma.

Based on the USP39 gene knockdown expression profile in U87 cells, ADAM9 gene was screened as the target molecule of USP39. ADAM9, also known as meltrin γ or metalloprotease disintegrin cysteine‐rich protein‐9, was described as an 84‐kDa transmembrane cell surface protein [37]. ADAM9 influences the inflammation, developmental process, degenerative diseases and tumor biology. Moreover, increasing evidence has shown that ADAM9 plays an important role in the invasion and migration of tumor cells. For example, knockdown of ADAM9 expression decreased lung cancer metastases to the brain [38], RNA interference to ADAM9 attenuated the invasiveness of Tenascin‐C‐stimulated brain tumor‐initiating cells [39] and ectopic expression of ADAM9 abolished microRNA (miR)‐1272‐induced inhibition of glioma cell migration [40]. Mechanistically, integrin β1 was demonstrated to be a molecular target of ADAM9 to regulate the migration and invasion of cells. Integrin not only could physically tether cells to the matrix, but also send and receive molecular signals that regulate the processes of cell invasion and migration [41]. ADAM9 interacted with integrin β1 and regulated its endocytosis [20, 42, 43]. In ADAM9‐silenced prostate cancer cells, the expression levels of integrin β1 was up‐regulated, whereas the activity of integrin β1 at cell surface was damaged and the formation of focal adhesions was delayed, probably explaining the reduction in cell adhesion and migration [43]. Therefore, to demonstrate further that ADAM9 acts as the target molecule of USP39 in the regulation of glioma cell migration and invasion, we examined the expression levels of integrin β1 in the shUSP39‐U251 cells *in vitro* and *in vivo*. Interestingly, in the shUSP39‐U251 cells and tumors, even though the expression levels of integrin β1 were upregulated, its molecular weight was obviously lower than that in shNC‐U251 cells. According to the technical file of antibody against integrin β1 and other studies, the precursor integrin β1 was 115 kDa, whereas the activated and mature integrin β1 was 135 kDa [44, 45]. Thus, in the shUSP39‐U251 cells, the downregulated ADAM9 induced the high expression of inactivated precursor integrin β1 on the cell surface, causing the reduced migration and invasion of cells. Correspondingly, in the ov‐USP39 U87 tumor, the expression levels of precursor integrin β1 were obviously lower than that in the ov‐Con U87 tumor. These results indicated that USP39 promotes the migration and invasion of glioma cells, at least in part, depending on its regulation of ADAM9 and integrin β1 expression.

An essential remaining question is how USP39 upregulates ADAM9 mRNA and protein levels. USP39 was reported to act as a splicing factor and to regulate the maturation of the mRNA of certain genes, such as *Aurora B* [8], *RB1* [10], *EGFR* [11], *FoxM1* [12] and *TAZ* [18]. Although the abnormal expression of ADAM9 mRNA and protein has been investigated in glioma [27], the mechanisms controlling the processing of ADAM9 mRNA are still little known. To detect precursor and mature ADAM9 mRNA, two primer pairs were specifically designed for a region of the longest ADAM9 transcript; we found that the ratio of spliced to unspliced ADAM9 mRNA was decreased or increased in the glioma cells with USP39 siRNA or overexpressed plasmid, respectively. This method has been previously employed to examine the splicing rate of multiple gene transcripts [11, 46]. The RIP and *in vitro* splicing assays were executed to confirm further the direct function of USP39 on the regulation of ADAM9 pre‐mRNA maturation. As USP39 has been demonstrated to take part in the processing of many gene mRNA, it is reasonable to speculate that the roles of USP39 on mRNA splicing are not exclusive to ADAM9 mRNA.

## 5 Conclusion

In summary, USP39 promotes human glioma migration and invasion by inducing ADAM9 mRNA maturation. This study provides a new mechanism of effect of USP39 and reveals its pivotal involvement in the migration and invasion of human glioma. Our results suggest that USP39 could be a target for the development of novel treatment approaches for glioma.

## Conflict of interest

The authors declare no conflicts of interest.

## Author contributions

YX and WC conceived and designed the study. YX and WM performed the experiments and completed the paper. WH provided the human glioma TMA. YX, QD, XZ, XM, PS, HW and XC analyzed the data. YX, ZW and WC reviewed and edited the manuscript. All authors read and approved the final manuscript.

### Peer Review

The peer review history for this article is available at https://publons.com/publon/10.1002/1878‐0261.12958.

## Supporting information


**Fig. S1**. The expression levels of proteins in tri‐snRNP complex in human glioma.

**Fig. S2**. Kaplan–Meier curve showing the 17‐year survival rate of The Cancer Genome Atlas (TCGA) samples classified by low or high expression of the genes of tri‐snRNP complex.

**Fig. S3**. The expression correlation of USP39 and other genes of the tri‐snRNP complex in glioma.

**Fig. S4**. The expression levels of USP39 in U251 and U87 cells.

**Fig. S5**. Gene ontology analysis of the differentially expressed transcripts in U87 cells expressing USP39 shRNA.

**Fig. S6**. Scatterplots of the correlation analysis between USP39 and CDH7, MMP27, PCDH11X, MMP16, ADIPOR1, CAMSPA1, ADAMTS10 expression of TCGA glioma samples from GEPIA databases.

**Fig. S7**. The images of U87 cells transfected with GFP‐tagged ADAM9.

**Fig. S8**. Silencing ADAM9 rescues the improved abilities of migration and invasion of U87 cells induced by USP39 overexpression.

**Fig. S9**. The effect of USP39 on the ubiquitination of ADAM9.

**Fig. S10**. Downregulated USP39 expression enhances the protein levels of integrin β1.

**Table S1**. The characteristics of the paraffin‐embedded samples for IHC analysis.

**Table S2**. Twenty‐one genes differentially expressed in the U87‐shNC cells and U87‐shUSP39 cells.

**Table S3**. The characteristics of the fresh human glioma samples for western blotting analysis.

## Data Availability

The gene expression microarray data that support the findings in this study have been deposited in NCBI GEO and are accessible through GEO Series accession number GSE159823. All data generated or analyzed during this study are included in this published article and its supplementary information files.
