# Supplementary figures and images for: Ubiquitin‐specific peptidase 39 promotes human glioma cells migration and invasion by facilitating ADAM9 mRNA maturation
Source: Mol Oncol. 2021 May 2;16(2):388–404. doi: 10.1002/1878-0261.12958 (PMC8763660; doi:10.1002/1878-0261.12958)

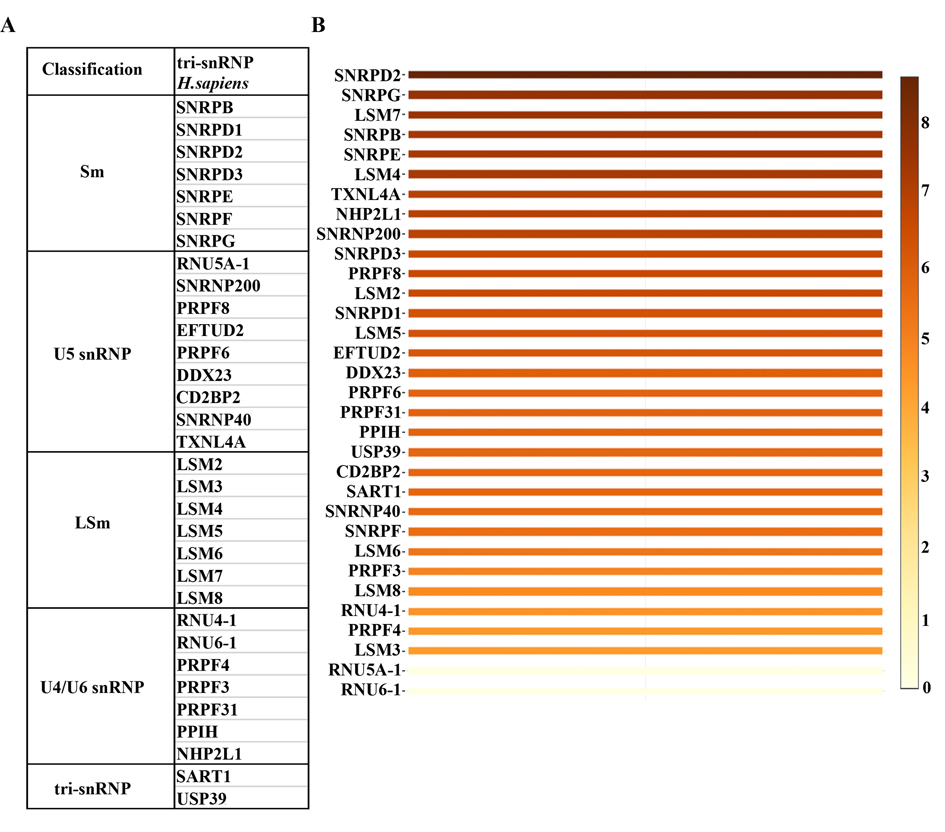

Supplement: Supplementary file 1 — Fig. S1. The expression levels of proteins in tri‐snRNP complex in human glioma. [file MOL2-16-388-s003.jpg]

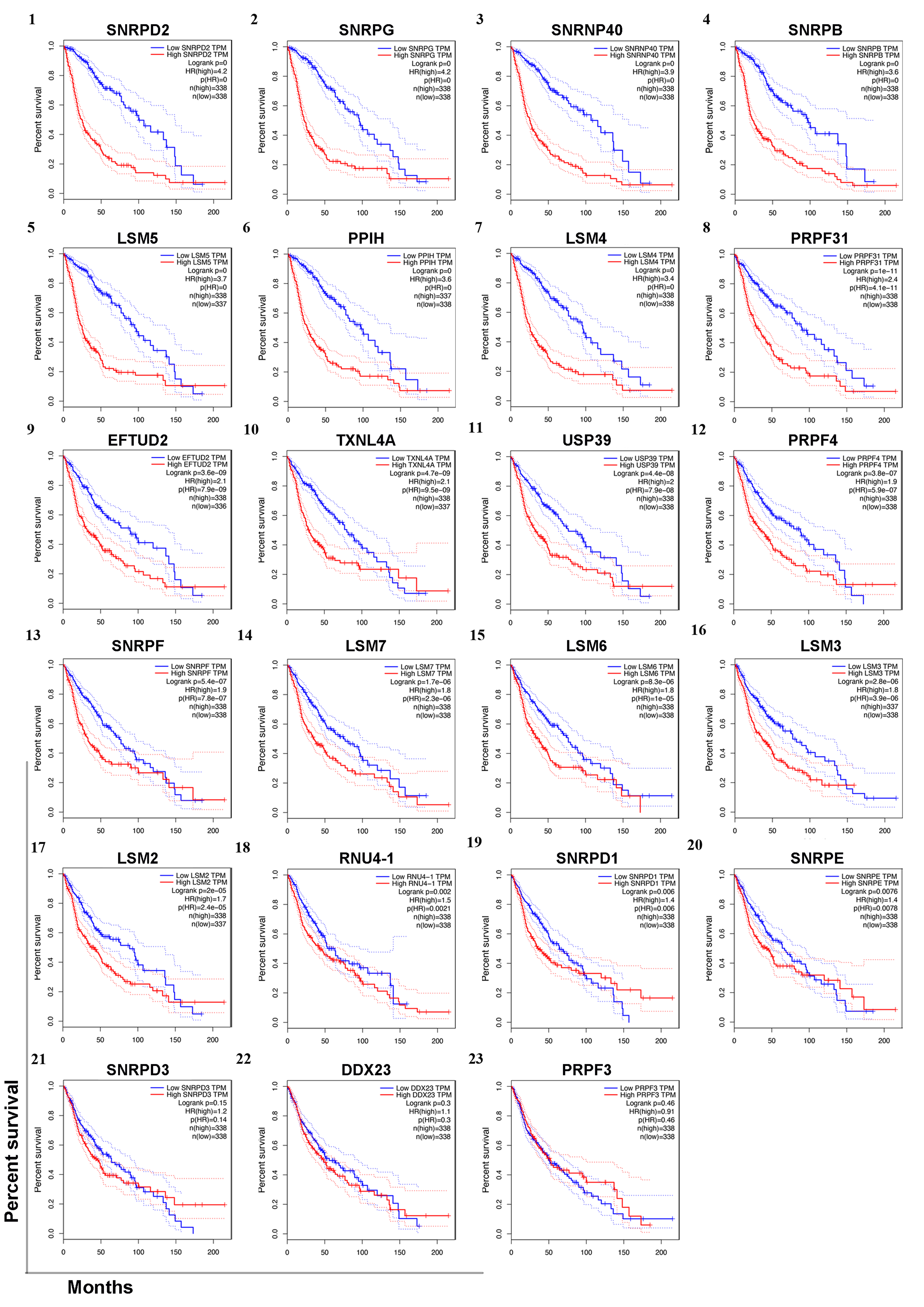

Supplement: Supplementary file 2 — Fig. S2. Kaplan–Meier curve showing the 17‐year survival rate of The Cancer Genome Atlas (TCGA) samples classified by low or high expression of the genes of tri‐snRNP complex. [file MOL2-16-388-s013.jpg]

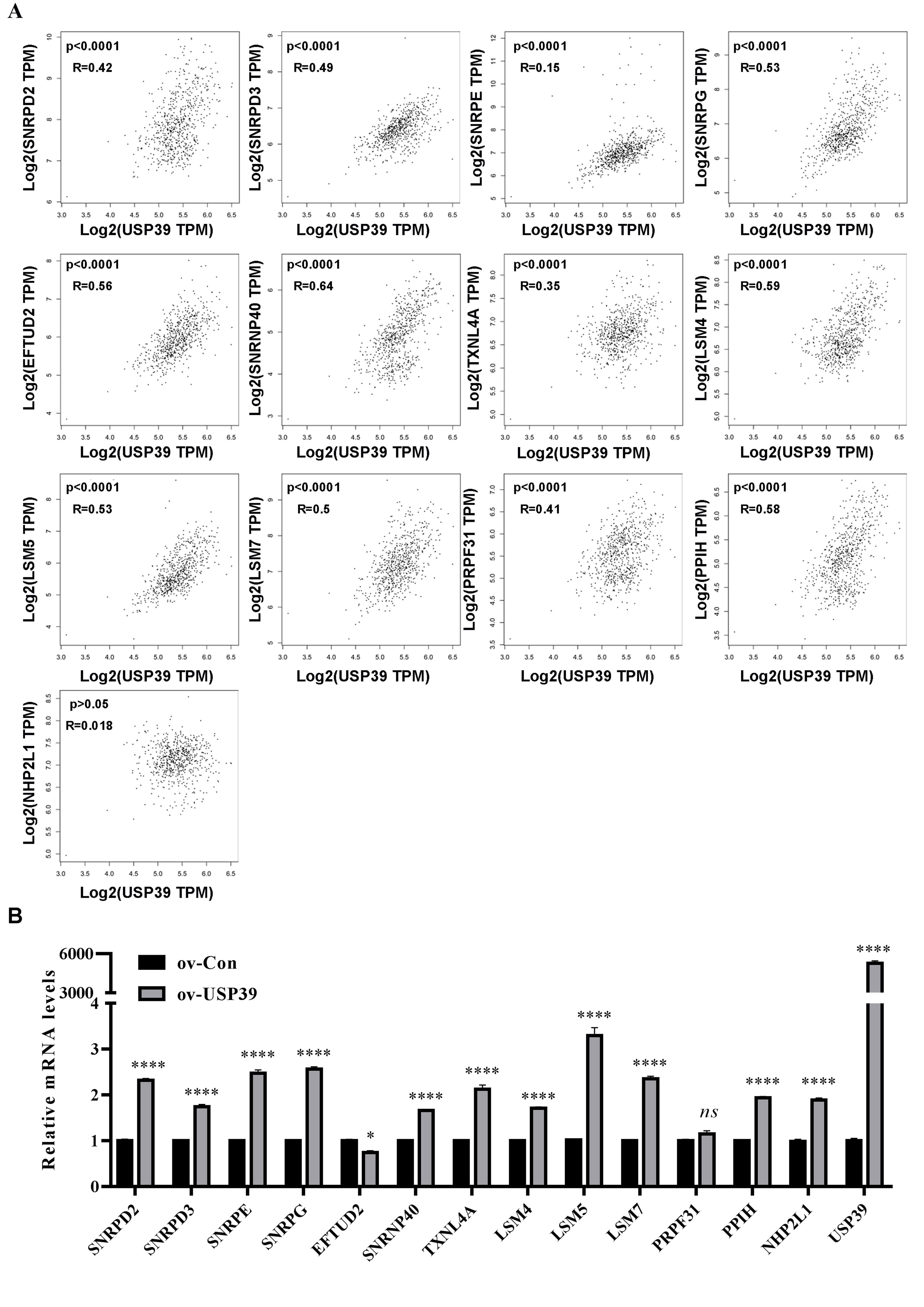

Supplement: Supplementary file 3 — Fig. S3. The expression correlation of USP39 and other genes of the tri‐snRNP complex in glioma. [file MOL2-16-388-s002.jpg]

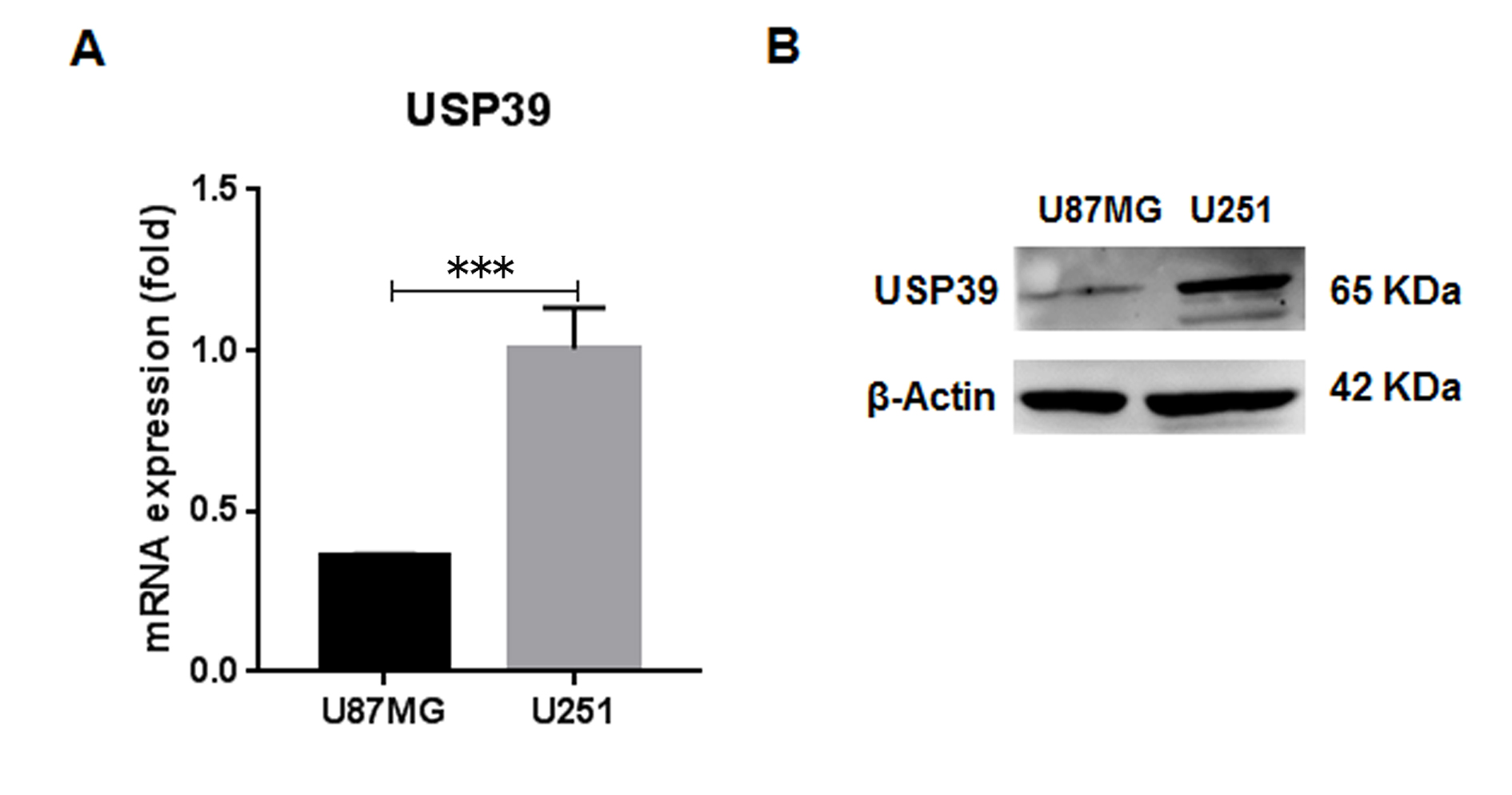

Supplement: Supplementary file 4 — Fig. S4. The expression levels of USP39 in U251 and U87 cells. [file MOL2-16-388-s012.jpg]

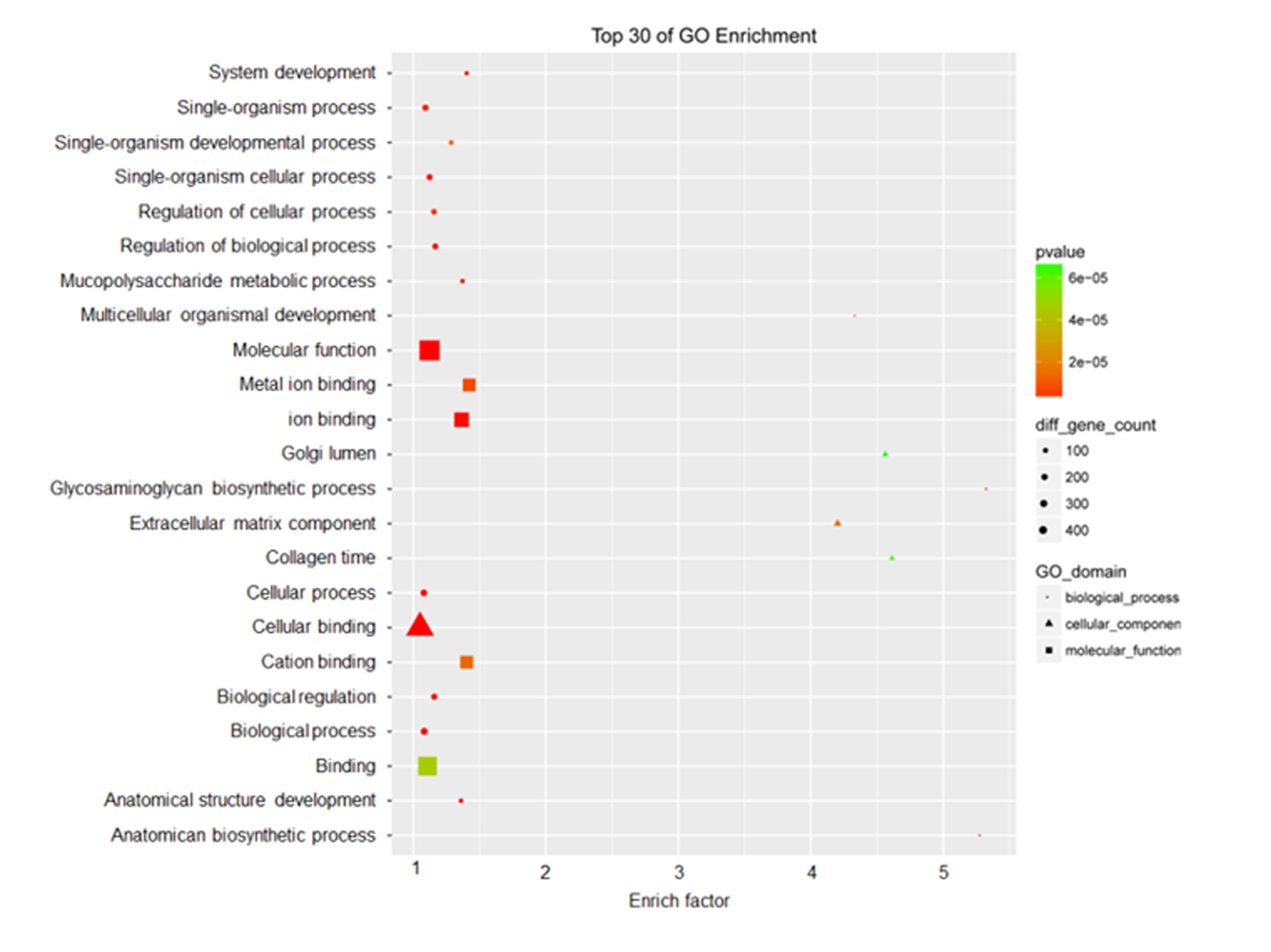

Supplement: Supplementary file 5 — Fig. S5. Gene ontology analysis of the differentially expressed transcripts in U87 cells expressing USP39 shRNA. [file MOL2-16-388-s001.jpg]

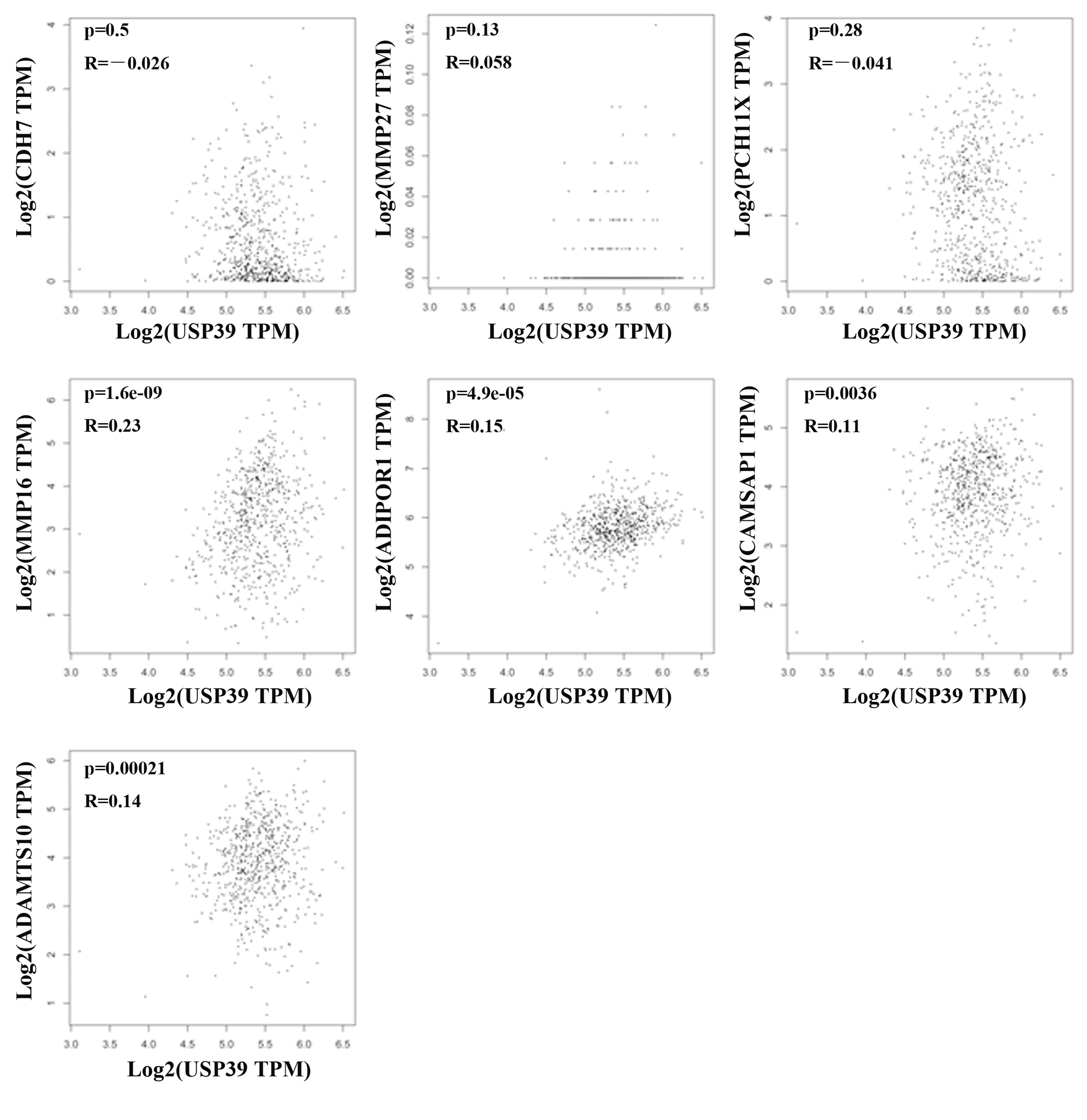

Supplement: Supplementary file 6 — Fig. S6. Scatterplots of the correlation analysis between USP39 and CDH7, MMP27, PCDH11X, MMP16, ADIPOR1, CAMSPA1, ADAMTS10 expression of TCGA glioma samples from GEPIA databases. [file MOL2-16-388-s009.jpg]

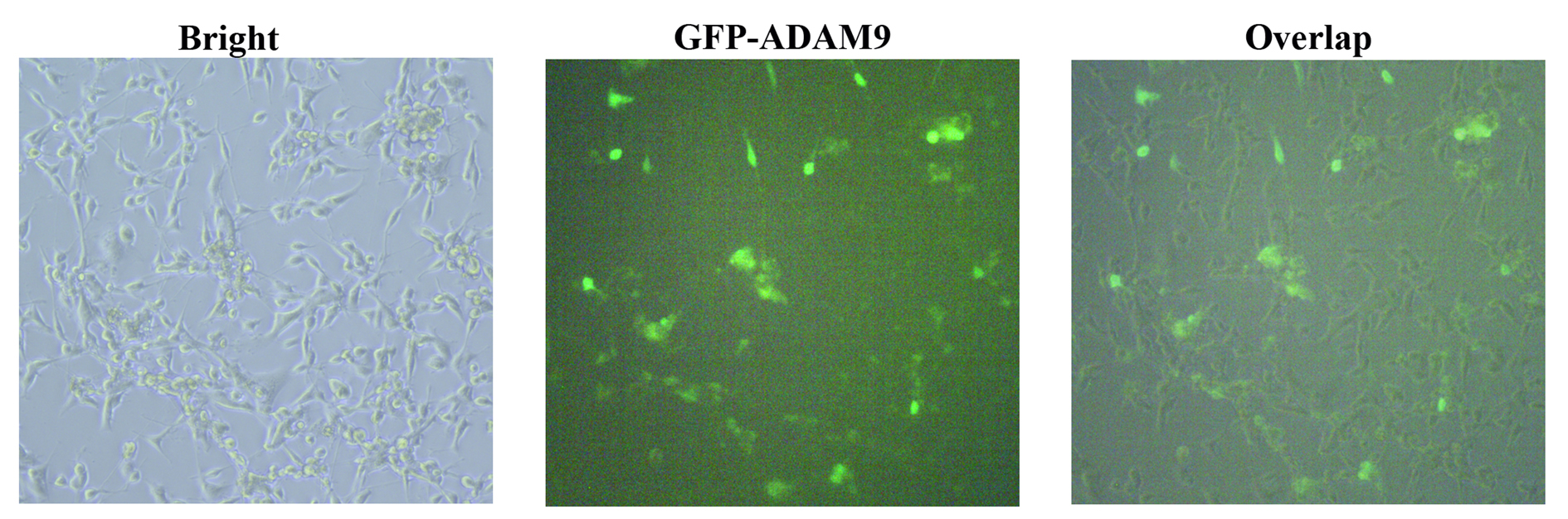

Supplement: Supplementary file 7 — Fig. S7. The images of U87 cells transfected with GFP‐tagged ADAM9. [file MOL2-16-388-s010.jpg]

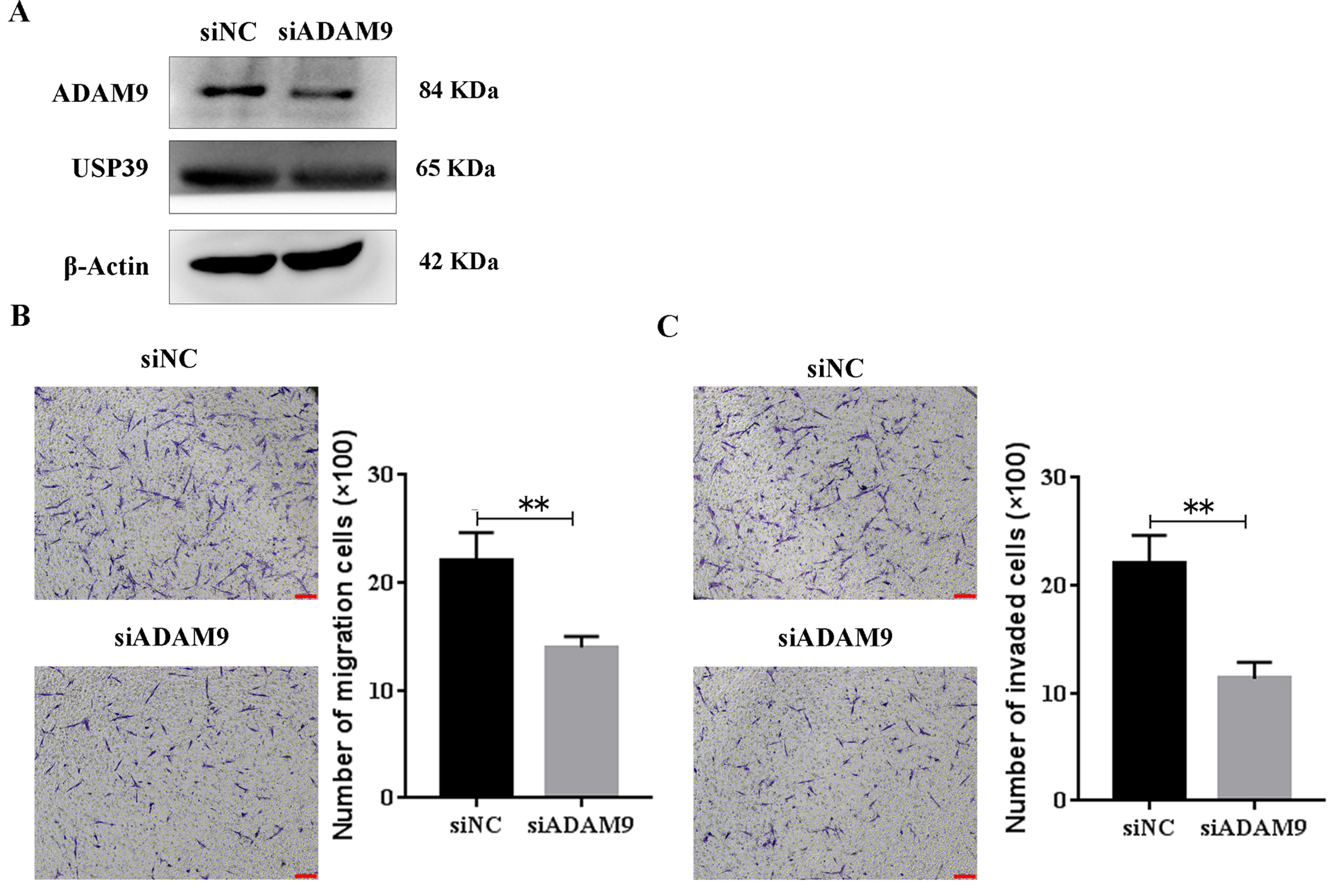

Supplement: Supplementary file 8 — Fig. S8. Silencing ADAM9 rescues the improved abilities of migration and invasion of U87 cells induced by USP39 overexpression. [file MOL2-16-388-s005.jpg]

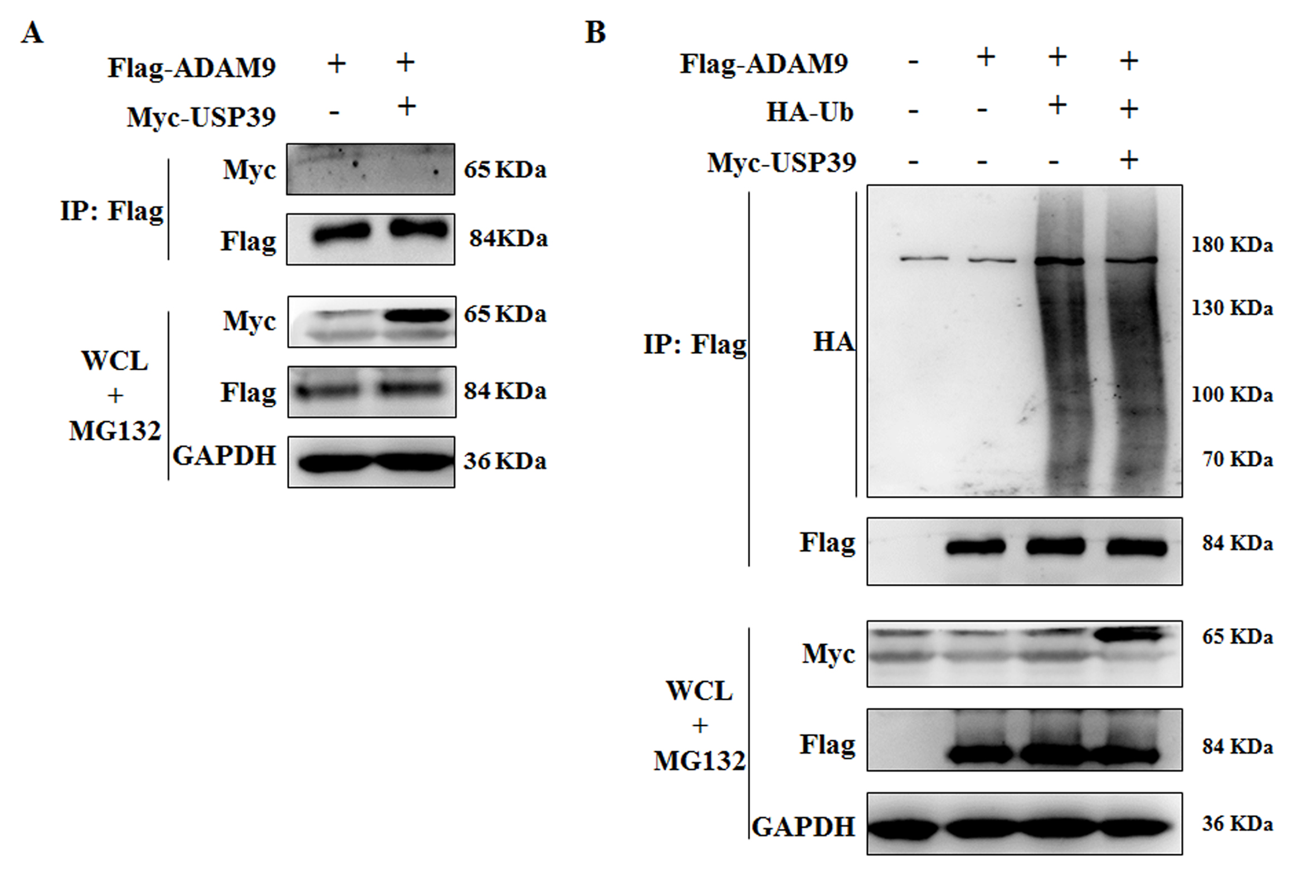

Supplement: Supplementary file 9 — Fig. S9. The effect of USP39 on the ubiquitination of ADAM9. [file MOL2-16-388-s007.jpg]

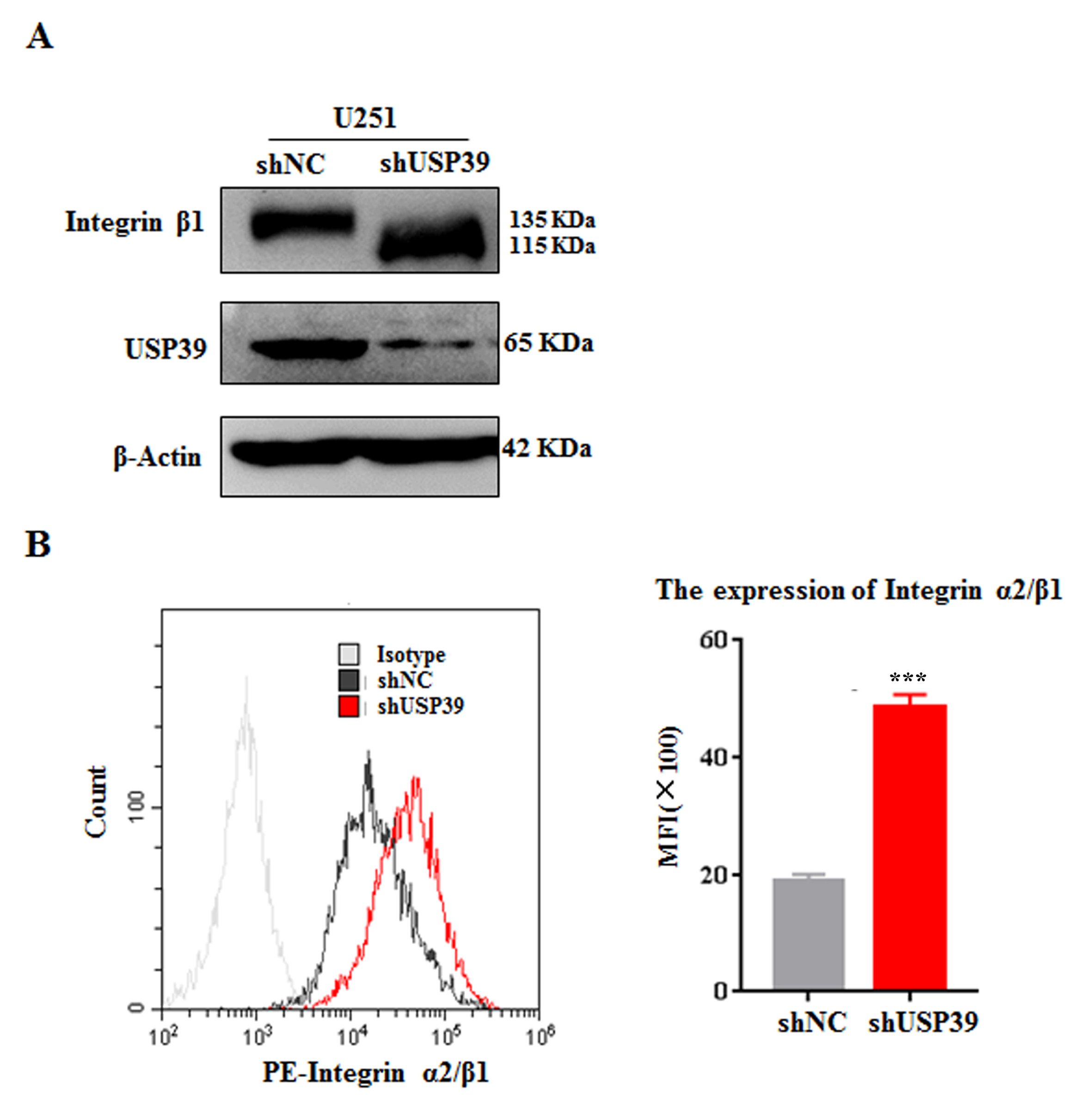

Supplement: Supplementary file 10 — Fig. S10. Downregulated USP39 expression enhances the protein levels of integrin β1. [file MOL2-16-388-s004.jpg]

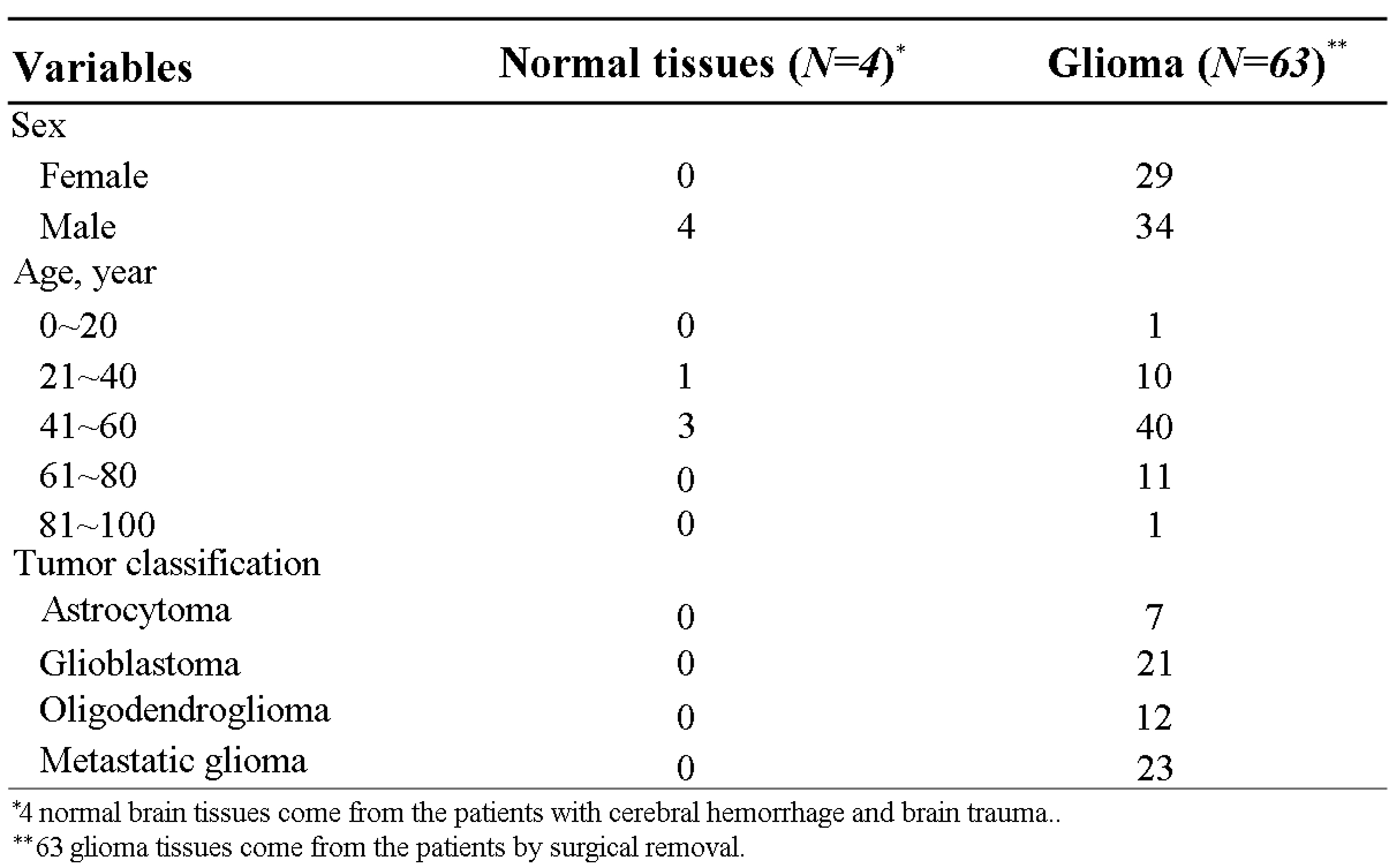

Supplement: Supplementary file 11 — Table S1. The characteristics of the paraffin‐embedded samples for IHC analysis. [file MOL2-16-388-s008.jpg]

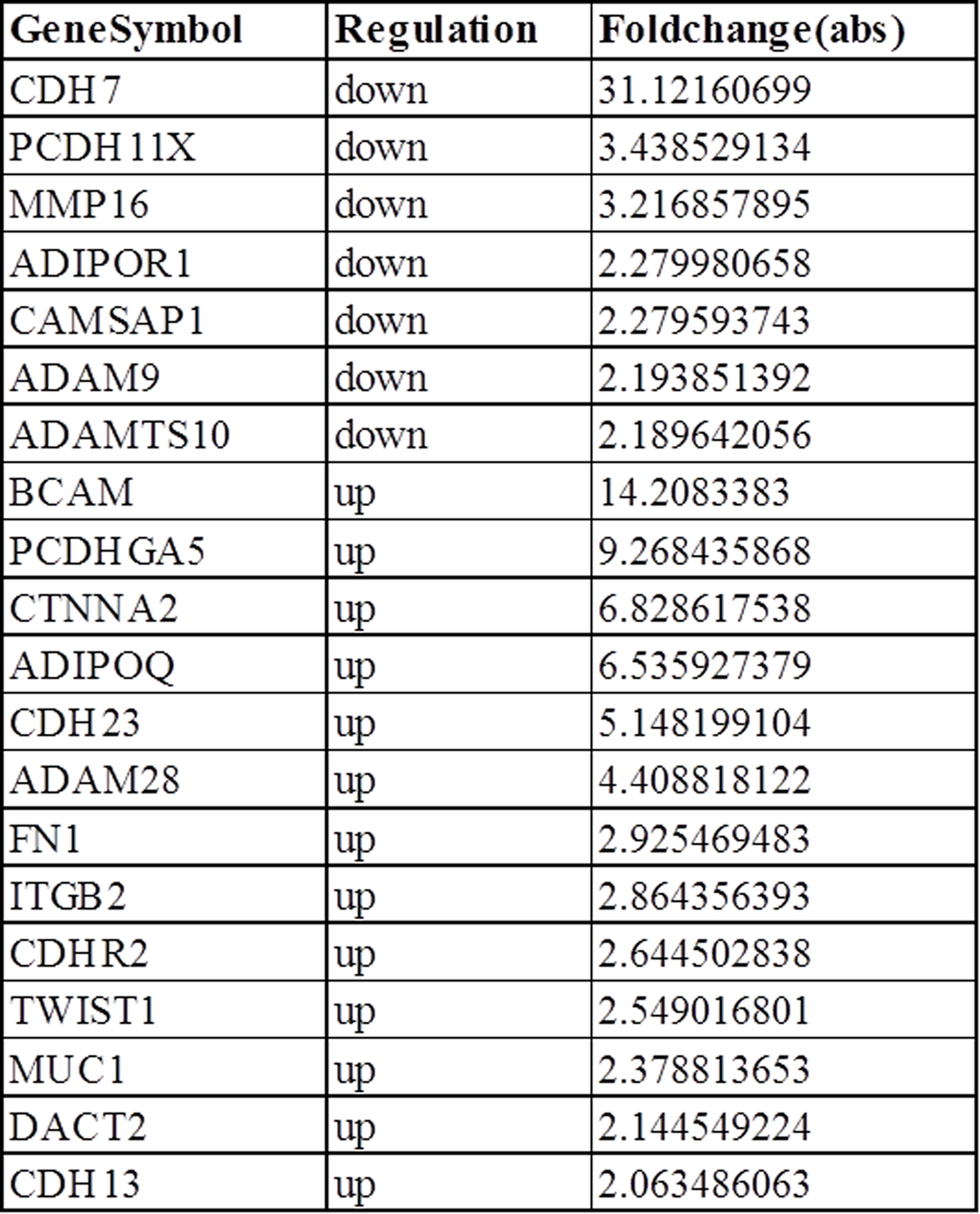

Supplement: Supplementary file 12 — Table S2. Twenty‐one genes differentially expressed in the U87‐shNC cells and U87‐shUSP39 cells. [file MOL2-16-388-s011.jpg]

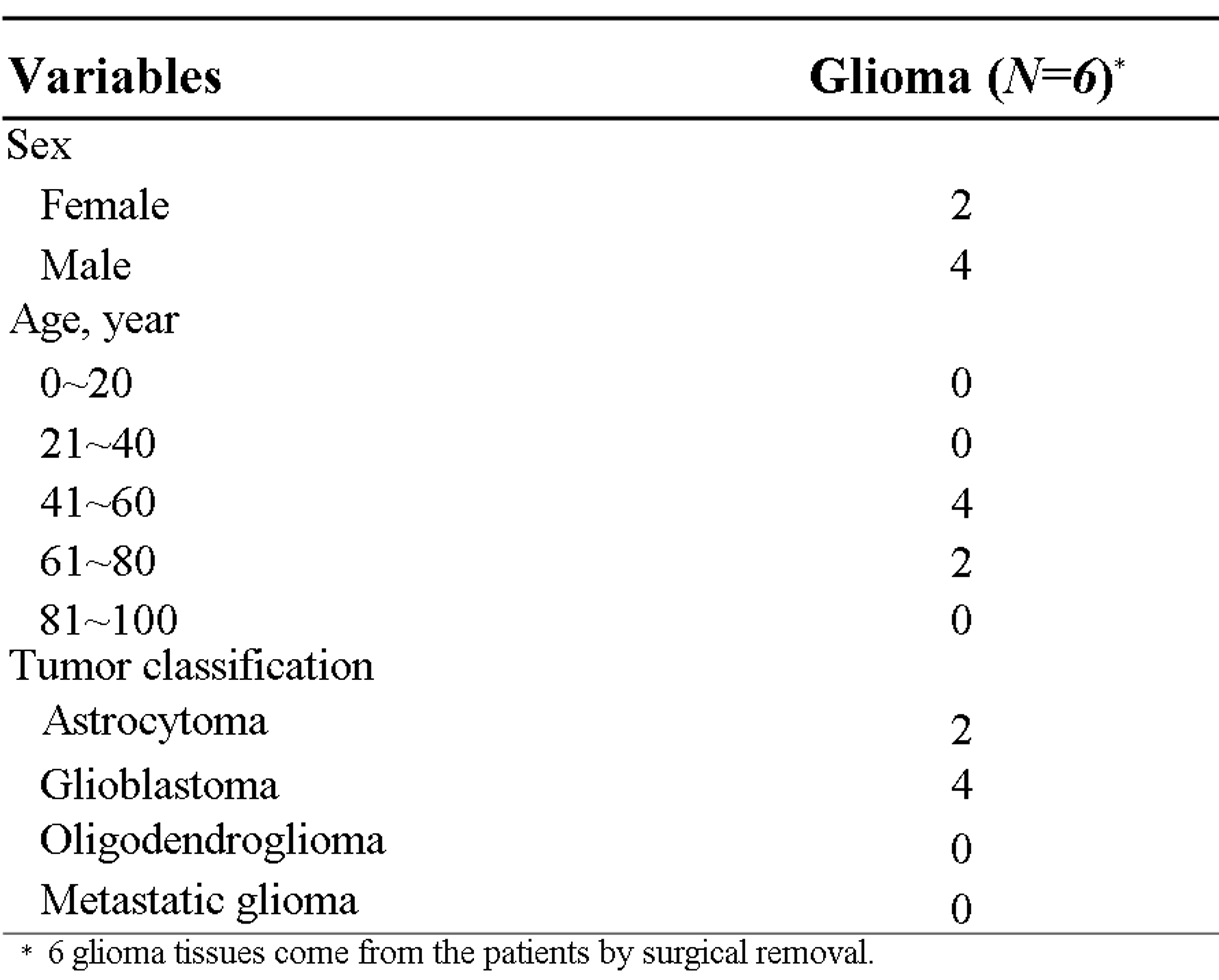

Supplement: Supplementary file 13 — Table S3. The characteristics of the fresh human glioma samples for western blotting analysis. [file MOL2-16-388-s006.jpg]
